# Inhibition of the mitochondrial protein Opa1 curtails breast cancer growth

**DOI:** 10.1186/s13046-022-02304-6

**Published:** 2022-03-12

**Authors:** Margherita Zamberlan, Amandine Boeckx, Florian Muller, Federica Vinelli, Olivier Ek, Caterina Vianello, Emeline Coart, Keitaro Shibata, Aurélie Christian, Francesca Grespi, Marta Giacomello, Ingrid Struman, Luca Scorrano, Stéphanie Herkenne

**Affiliations:** 1 Department of Biology, University of Padova, Via U. Bassi 58B, 35121 Padova, Italy; 2 Veneto Institute of Molecular Medicine, Via Orus 2, 35129 Padova, Italy; 3 Laboratory of molecular angiogenesis, GIGA-Research, Avenue de l’Hôpital, 1, 4020 Liège, Belgium

## Abstract

**Background:**

Mitochondrial fusion and fission proteins have been nominated as druggable targets in cancer. Whether their inhibition is efficacious in triple negative breast cancer (TNBC) that almost invariably develops chemoresistance is unknown.

**Methods:**

We used a combination of bioinformatics analyses of cancer genomic databases, genetic and pharmacological Optic Atrophy 1 (OPA1) inhibition, mitochondrial function and morphology measurements, micro-RNA (miRNA) profiling and formal epistatic analyses to address the role of OPA1 in TNBC proliferation, migration, and invasion in vitro and in vivo*.*

**Results:**

We identified a signature of OPA1 upregulation in breast cancer that correlates with worse prognosis. Accordingly, OPA1 inhibition could reduce breast cancer cells proliferation, migration, and invasion in vitro and in vivo. Mechanistically, while OPA1 silencing did not reduce mitochondrial respiration, it increased levels of miRNAs of the 148/152 family known to inhibit tumor growth and invasiveness. Indeed, these miRNAs were epistatic to *OPA1* in the regulation of TNBC cells growth and invasiveness.

**Conclusions:**

Our data show that targeted inhibition of the mitochondrial fusion protein OPA1 curtails TNBC growth and nominate OPA1 as a druggable target in TNBC.

**Supplementary Information:**

The online version contains supplementary material available at 10.1186/s13046-022-02304-6.

## Background

Breast cancer is the second cause of cancer death and the most frequently diagnosed tumor in women. Breast cancer is a highly heterogeneous disease. Several subtypes are classified by their estrogen (ER) or progesterone (PR) receptor and HER2 (Human epidermal growth factor receptor 2) immunochemistry positivity and respond differently to treatment [1]. Progresses in early detection, diagnosis and therapy greatly improved breast cancer clinical outcome, resulting in a steep decline in mortality rate [2]. Nevertheless, some subtypes remain difficult to treat.

Triple negative breast cancer (TNBC) constitutes the most aggressive breast cancer subtype and is highly heterogeneous. Different classifications of TNBC are available, based on gene expression profile. For example, TNBC can be classified in basal-like 1 and 2 (BL1 and BL2), mesenchymal (M), mesenchymal stem-like (MSL), immunomodulatory (IM), and luminal androgen receptor (LAR) [3]; or in four classes: M that expresses growth factor receptors, LAR overexpressing AR (Androgen Receptor), basal-like immune-activated (BLIA) and basal-like immunosuppressed (BLIS) [4]. Finally, TNBC can be classified in subtypes based on cluster analysis of gene expression. However, gene expression analysis is not commonly available in clinical practice [5]. Because of the poor response of TNBC to therapy, refinements in its classification are crucial to develop targeted therapies [6]. For example, a target for the LAR subtype is the androgen receptor; PI3K/AKT/mTOR, MET, TGF-beta, and NOS pathways are targets for the M subtype. For TNBC with PDL-1 positive immune cells, chemotherapy and immunotherapy can favor the response to immune checkpoint inhibitors. For the basal-like subtypes, a novel approach consists in the inhibition of poly-ADP ribose polymerase (PARP) in combination with platinum-based chemotherapy, or eventually with PI3K inhibitors in patients insensitive to PARP inhibitors alone [7, 8]. Despite these advances, TNBC is not currently treated with targeted drugs. Conventional therapy is employed, with anthracyclines and taxanes being widely used [9]. However, resistance to chemotherapy almost invariably develops and TNBCs remain the most challenging cancer in terms of therapy, calling for the identification of novel liabilities that can be targeted.

The emergence of chemoresistance in breast cancer is a complex process. One facet of chemoresistance is the ability of cancer cells to adapt their metabolism to match nutrient availability. Specific metabolic phenotypes allow TNBCs to sustain proliferation, progression, metastatization and resistance to chemotherapy [10–12]. Molecularly, gene amplification and mutations in MYC, p53, BECN1, PTEN, PIK3CA and RB1 appear as key regulators in the metabolic reprogramming of TNBCs [13, 14]. Like most other cancers, TNBCs rely on increased glucose uptake and glycolysis that are boosted by transcription factors (such as HIF-1α, NRF2, C-MYC) driving overexpression of glycolytic enzymes (such as HK2, PKM2, LDHA). Glycolysis is thus a potential therapeutic target, but effective treatments curtailing glycolytic metabolism have not yet reached clinical practice [15].

TNBC can rely also on oxidative metabolism [16]. Interestingly, mitochondrial Oxidative Phosphorylation (OXPHOS) can promote TNBC migration and metastasis dissemination [17]; conversely, lack of mtDNA reduces the tumorigenic phenotype and growth of breast cancer cells, making them more sensitive to cytotoxic drugs [18]. Unfortunately, inhibition of mitochondrial respiration is not a viable therapeutic strategy for chemoresistant TNBC, given the predicted severe side effects of OXPHOS inhibition. Mitochondrial function is however influenced by mitochondrial shape and morphology [19], offering a potential target to modulate mitochondrial respiration without directly inhibiting OXPHOS. Mitochondrial morphology depends on the balance between fusion and fission processes. Fission is controlled by dynamin-related protein 1 (DRP1) that binds to its adaptors on the outer mitochondrial membrane (OMM), fission 1 (Fis1), mitochondrial fission factor (MFF), and mitochondrial division (Mid) 49 and 51. Fusion is controlled by mitofusins (MFN) 1 and 2 in the OMM and by optic atrophy protein 1 (OPA1) in the inner mitochondrial membrane (IMM) [20, 21]. OPA1 is an attractive candidate in the context of targeted mitochondrial cancer therapy [22], based on clinical and biological features of this mitochondrial protein. First, in addition to its core function in mitochondrial fusion, OPA1 regulates several hallmarks of cancer: cytochrome c release and apoptosis, mitochondrial respiration, cell proliferation and angiogenesis [19, 21–24]. Second, *OPA1* is often amplified across pan-cancer genomic datasets, including breast [25]. Third, *OPA1* overexpression correlates with poor prognosis and reduced sensitivity toward chemotherapy [26–28]. Finally, in acute myeloid leukemias (AML) resistant to the Bcl-2 antagonist venetoclax, OPA1 inhibition restores sensitivity to venetoclax [29].

Here we set out to investigate if OPA1 can be a therapeutic liability of breast cancer. Our data indicate that breast cancer prognosis negatively correlates with OPA1 levels and that OPA1 genetic and pharmacological inhibition curtails breast cancer in vitro and in vivo by impinging on miRNAs of the 148/152 family.

## Materials and methods

### Mouse models

Animal housing and all the experimental procedures were authorized by the Belgian ethical committee (Protocol number: 2166). All mice used were bred in the NOD SCID background. Mice were housed 4 per cage in a temperature (22–24 °C) controlled colony room, maintained on a 12-h light/dark cycle (07:00 to 19:00 light on), with standard food and water provided ad libitum and environmental enrichments.

For tumor experiments, we used 6-week-old females per group, and we repeated each experiment twice. The allocation of mice in different group was random. During experiments, mice were monitored twice per day. Behavior, weight, furrow, and tumor size were controlled every day. The total number of mice analyzed for each experiment is detailed in the figure legends. All in vivo experiments were approved by the local Animal Ethic Commission (File number 2166, “Commission d’éthique de l’utilisation des animaux de l’Université de Liège”).

### Cell culture

All cells were grown at 37 °C in a 5% CO_2_ humid atmosphere. MDA-MB-231 breast adenocarcinoma cells (ATCC-HTB26; sex: F), MCF7, HS578T and T47D (kindly p-rovided by Dr. Gilles, University of Liège) were cultured in DMEM containing 1 g/L glucose, supplemented with 10% fetal bovine serum (FBS), 100 U/ml penicillin and 100 μg/ml streptomycin.

### Generation of MDA-MB-231 *OPA1*^−/−^ cells

MDA-MB-231 *OPA1*^−/−^ cells were generated by CRISPR/Cas9. Using the CRISPR Design Tool at crispr.mit.edu we designed the following guide RNAs targeting Exon 2 of OPA1:

- Oligo1: 5′-CACCGAGTCTCGTAGCTAATCTTGC-3′
- Oligo2: 5′-AAACGCAAGATTAGCTACGAGACTC-3′

Oligos were annealed using T4 Polynucleotide kinase, ligated into the Cas9 from *S. pyogenes* with 2A-EGFP plasmid (PX458, Addgene Plasmid #48138) cutting at a BbsI restriction enzyme site. XL10-Gold ultracompetent bacteria were transformed and grown under ampicillin selection. Resulting colonies were picked and the insertion of the guide RNA’s were confirmed by sequencing. DNA was purified using a QIAprep Spin Minprep Kit (Qiagen) according to manufacturer’s instructions. The successful insertion of the guide RNAs into the vector was confirmed by the loss the restriction enzyme site for BbsI. The generated plasmids were transfected into MB-MDA-231 cells using Lipofectamine 3000 according to the manufacturer’s instructions. 48 h post-transfection, the GFP^+^ cells were single sorted by flow cytometry (FACS Aria, BD Biosciences). Single clones in 96-well plates were cultured and expanded in complete growth media and OPA1 levels were evaluated by western blot.

### Design of the synthetic miRNA

The miRNA mimics miR-148a-3p, miR-148b-3p, miR-152-3p and cel-miR-67 (control) are double-stranded RNAs designed using the method of Betancur et al. [30]. The mature miRNA strand was modified by the addition of phosphorylation at the 5′ end and the carrier strand was the complementary RNA sequence, carrying a two base 3′ overhang with mutations near the 3′ end to thermodynamically destabilize the strand and induce faster degradation. Oligonucleotides were purchased from Eurogentec.

### MiRNA and siRNA transfection

Cells (18 × 10^5^ per well in a 6-wells plate; 8 × 10^5^ per well in a 48-wells plate and 8 × 10^4^ per well in a 96-wells plate) were transfected with the following siRNAs: a non-relevant human sequence (*UNREL*) (AM4635 Ambion, 20 nM), *OPA1* (144,409 Invitrogen, 20 nM), *OPA1* siRNA2 (36,409, Invitrogen 20 nM), *MFN1* (50 nM) and *MFN2* (50 nM) using Dharmafect-4 (Dharmacon) according to manufacturer’s instructions. All transfection experiments described in this paper were carried out with *MFN1* and *MFN2* siRNA1. Similar results were obtained with *MFN1* and *MFN*2 siRNA 2 (data not shown). Functional assays on transfected cells and evaluation of transfection efficiency were performed after 72 h. Cells were trypsinized, stained with trypan blue and living cells were seeded for the different functional assays.

For miRNAs transfection, pre-miR-148a, 148b and 152 and anti-miR-148a, 148b and 152 and the non-relevant human sequences (P-UNREL for the pre-miR-control and A-UNREL for the anti-miR-control) were used at a concentration of 10 nM following the same protocol as siRNAs transfection.

### Plasmid transfection

30 × 10^5^ cells were seeded on 6-well plate and the transfections were performed using Lipofectamine 3000 according to the manufacturer’s instructions. For transient overexpression of Opa1, MDA-MB-231 were transfected with pMSCV-Opa1 [21] and the assays performed after 48 h.

### Cell proliferation assay

Cells in a 96-well plate were transfected in 100 μl DMEM 1 g/L glucose for 72 h. Medium was changed with DMEM supplemented with 10% FCS for 24 h to induce proliferation. The thymidine analogue 5-bromo-2-deoxyuridine (BrdU) was added and incubated for 16 h. BrdU incorporation was measured with the Cell Proliferation ELISA BrdU chemiluminescence kit (Roche Applied Science) according to the manufacturer’s protocol. Where indicated, cells were treated with MYLS22 (50 μM) or DMSO when the complete DMEM medium was added.

### Scratch wound migration assay

Cells in 48-wells plates were transfected in 800 μL of DMEM 1 g/L glucose for 72 h and the head of a 200 μL tip was used to perform a wound. Migration of cells into the wound was measured 6 h later. The percentage of coverage was calculated using the following formula:

$$\%\mathrm{Coverage}\:=\:\lbrack({\mathrm{Width}}_{\mathrm T0}-{\mathrm{Width}}_{\mathrm T6})/{\mathrm{Width}}_{\mathrm T0}\rbrack\ast100$$

Where indicated, cells were treated with MYLS22 (50 μM) or DMSO when the scratch was performed.

### Boyden chamber assay

Cells were seeded on the upper wells of 8-μm Boyden chambers (24-wells Transwell, Costar Corp) and incubated for 16 h in in 300 μl DMEM without FCS. To induce migration, the lower chamber was filled with 600 μl of DMEM supplemented with 10% FCS. Cells were allowed to migrate for 6 h at 37 °C. The membrane insert was removed and flipped so that the side towards the lower chamber faced the operator. Cells were fixed for 10 min in MeOH (100% V/V, ice cold) and stained with 4% Giemsa and the insert was mounted on microscope slides. Cells were imaged using an Olympus CKX41 microscope (Olympus Life Science) and counted using ImageJ (NIH). Where indicated, the media in the upper chamber was supplemented with MYLS22 (50 μM) or DMSO when migration was induced.

### Cell adhesion assay

Cells (3 × 10^4^) were seeded on Fibronectin- or Gelatin-coated 96-wells plate for 1 h, washed with PBS and fixed for 10 min in MeOH (100% V/V, ice cold). Cells were then stained with Crystal violet solubilized in acetic acid for 15 min and washed three times. Crystal violet absorbance was measured at 520 nM. Where indicated, cells were pretreated with MYLS22 (50 μM) or DMSO 24 h before the assay.

### Invasion assay

Spheroids were prepared as previously described [31]. Homospheroids composed of transfected MDA-MB-231 were allowed to form in 96-well suspension plates for 48 h. Spheroids were then collected and seeded for 24 h in a 3D collagen matrix with culture medium and methylcellulose. To quantify invasion of collagen matrix by MDA-MB-231 cells, wells were imaged using an Olympus CKX41 microscope (Olympus Life Science) and the areas of invasion and of the spheroids was calculated using ImageJ (NIH). The invasion rate was determined as the ratio of the area of invasion over the area of the spheroid.

### Apoptosis assay

WT and MDA-MB-231 *OPA1*^−/−^ cells (1 × 10^5^) were grown in 6-wells plates. After 24 h cells were stained with Annexin-V-FICT and propidium iodide (PI), according to manufacturer’s protocol (eBioscience™). The rate of cell death, expressed as percentage of the Annexin-V-positive events in the total population, was measured by flow cytometry (FACS Calibur, BD Biosciences). Apoptosis was determined as described above in transfected MDA-MB-231 cells that were transferred to the 6 wells plates 48 h after the transfection.

### Electron microscopy

Cells grown in 24-wells plates were fixed for 1 h at 4 °C with freshly prepared 2.5% (V/V) glutaraldehyde in 0.1 M sodium cacodylate, pH 7.4. After washing with 0.1 M sodium cacodylate, cells were post-fixed in 1% OsO4, 1.5% K_4_Fe(CN)_6_ in 0.1 M sodium cacodylate pH 7.4, stained with 0.5% uranyl acetate, dehydrated in ethanol and embedded in Embed 812. Thin sections were imaged on a Tecnai-12 electron microscope (Philips-FEI) equipped with a Veleta (Olympus Imaging System) digital camera at the BioImaging Facility of the Dept. of Biology (University of Padua).

### Seahorse

Oxygen consumption rate (OCR) was measured with the Agilent Seahorse XFe24 Analyzers. WT, MDA-MB-231 *OPA1*^−/−^and transfected cells (3 × 10^6^) in complete DMEM were seeded in XF24 cell culture microplates, resulting in 60–70% confluency. After 24 h, the medium was replaced with XF medium (DMEM 1 g/L glucose, 0.58 g/L Glutamine, 1 mM Sodium Pyruvate, 0.015 g/L Phenol Red, pH 7.2) and plates were transferred to a 37 °C incubator not supplemented with CO_2_ for 1 h to equilibrate temperature and pH. After three measurements of basal OCR, 70 μl of solutions containing oligomycin, FCCP or rotenone and antimycin A were sequentially added to each well to reach final concentrations of 1 μM oligomycin, 1 μM FCCP and 2 μM for rotenone and antimycin A. Following each compound injection, three measurements were acquired. OCR is expressed as pmol O_2_ per min.

### Mitochondrial membrane potential measurement

Cells were incubated with tetramethyl rhodamine methyl ester (TMRM, Invitrogen) were incubated at 37 °C for 30 min with 1 nM TMRM in the presence of 1 μg/ml cyclosporine H (CsH), a P-glycoprotein inhibitor. Where indicated, 2 μg/ml oligomycin and 2 μM FCCP were added for an additional 30 min. TMRM fluorescence was measured by flow cytometry (FACS Aria, BD Biosciences).

### ATP measurement

ATP levels in MDA-MB-231 cells were measured 72 h after transfection using ATPlite kit (Perkin Elmer) according to the manufacturer’s protocol.

### miRNAs extraction, cDNA production and gene expression

Total RNA was extracted using the miRNeasy kit (Qiagen) following manufacturer’s protocol. For Poly(A) tailing and reverse-transcription of miRNAs (BioLabs), 20 ng RNA was reverse transcribed into cDNA. The expression levels of miRNAs were analyzed using SYBR® Green I kit (Eurogentec) by 7900 Real-Time PCR System (Applied Biosystems) and the results were obtained with the 2^-ΔCt^ method [32]. The miRNA levels were normalized to RNU44 and RNU48.

### miRNA PCR array

MicroRNAs expression was evaluated using a Breast Cancer Focus microRNA PCR Panel, 96-well (Qiagen). Each plate contains 84 lyophilized LNA miRNA primer sets focusing on cancer-relevant human miRNAs, 3 reference genes (miRNAs and small nuclear RNAs, snRNAs), 3 inter-plate calibrators and 5 RNA spike-ins. According to manufacturer’s protocol,10 μl of the master mix (5 μl of cDNA previously synthetized, 500 μl SYBR Green, 50 μl of ROX Reference dye and 445 μl of nuclease-free water) was added in each well. qPCR was performed using the 7900 Real-Time PCR System (Applied Biosystems) according to the following conditions: 2 min at 95 °C then 40 cycles at 95 °C for 10 s and 56 °C for 1 min. The Qiagen tool Geneglob was used to analyze the data. The relative miRNA levels were normalized to global mean.

### MiRpaths analysis method

The DIANA-miRPath v3.0 analysis was used to determine the molecular pathways controlled by miRNAs 148a, 148b and 152 annotated on the Kyoto Encyclopedia of Genes and Genomes (KEGG), using the following default parameters: experimentally supported interactions from DIANA TarBase v.7.0; a *p*-value threshold of 0.05; and a microT threshold of 0.8. To reduce the number of false-positive miRNA targets, we applied a false discovery rate (FDR) correction to selected KEGG pathways. The algorithm used in this analysis was a one-tailed Fisher exact test.

### Analysis of gene expression by qRT-PCR

RNAs were extracted with the miRNeasy kit (Qiagen) according to manufacturer’s protocol. cDNA synthesis was performed with 1 μg total RNA and the iScript cDNA Synthesis Kit (BioRad) according to the manufacturer’s instructions. Resulting cDNAs (20 ng) were used for quantitative real-time PCR using the SYBR green method (Roche Applied Sciences). Thermal cycling was performed on an ABI Prism 7900 HT Sequence Detection System (Applied Biosystems). For all reactions, no template controls were run, and random RNA preparations were also subjected to sham reverse transcription to check for the absence of genomic DNA amplification. Quantitative real-time PCR was performed with SYBR green method (Bioline and ThermoFisher Scientific). Thermal cycling was performed on an Applied Biosystem 7900 HT detection system and a Stratagene MX3005P multiplex QPCR system (Applied Biosystems and Stratagene).

The relative transcript level of each gene was normalized to the housekeeping genes cyclophilin-A (PPIA), beta-2 microglobulin (B2M) and/or GAPDH. Primers were designed using Primer Express software and selected to span exon-exon junctions to avoid detection of genomic DNA (primer sequences are provided in supplementary Methods). Quantification of mRNA levels was calculated with the 2^-ΔCt^ method.

### Western blotting

Cells were lysed in lysis buffer and heated at 95 °C for 10 min. Equal amounts of protein were resolved by 8% SDS-PAGE (Biorad) and transferred to polyvinylidene fluoride membranes (PVDF) according to the manufacturer’s protocol. Membranes were blocked for 1 h at room temperature with 5% BSA (Sigma Aldrich) in Tris-buffered saline with 0.1% Tween 20 (TBS-T) and probed overnight at 4 °C with the indicated primary antibodies. After 3 washes with TBS-T, the appropriate secondary antibody at a 1:5000 dilution was added for 1 h at room temperature. The immunoreactive bands were visualized by enhanced chemiluminescence ECL kit (Pierce).

### Immunofluorescence

Cells (3 × 10^5^) were cultured on coverslips coated with gelatin (0.2%), washed with PBS, fixed with 4% paraformaldehyde for 15 min at room temperature, blocked, permeabilized with PBS containing 5%BSA and 0.5% saponin, and incubated overnight in PBS supplemented with 1% BSA and 0.1% saponin (PBS-BS, Sigma Aldrich) containing the indicated primary antibodies. After 3 washes with PBS cells were incubated with the appropriate secondary antibodies conjugated to Alexa Fluor 488 and 568 (Thermo Fisher Scientific) dissolved in PBS-BS for 1 h at room temperature. Samples were washed 3 times with PBS and coverslips were mounted on microscope slides using the DAPI containing ProlongFade (Invitrogen) mounting solution. Slides were placed on the stage of a LSM700 (Zeiss) confocal microscopy equipped with a 63X, Zeiss Plan-Apochromat 63x/1.4 Oil objective and excited using the appropriate laser line. Images were acquired using a 1048 × 1048 resolution with the ZEN software (Zeiss).

For immunofluorescence on tissues, 5 μm thick cryostat sections were fixed in acetone at − 20 °C and in 100% methanol at 4 °C and then incubated with rat monoclonal anti-PECAM-1 for 1 h at room temperature. Slides were washed and incubated with Alexa 488 anti-rat for 1 h at 4 °C. After three washes, slides were covered with coverslips in mounting medium (Victor Laboratories) and analyzed using a Leica DMI4000 epifluorescence microscope (equipped with 10X/0.25, 20X/0.35NA, 40X/0.6NA and 63X/1.25NA objectives).

### Orthotopic MDA-MB-231 adenocarcinoma implants

Subconfluent MDA-MB-231 *OPA1*^*−/−*^*:*OPA1 or MDA-MB-231 *OPA1*^*−/−*^ cells were trypsinized, washed and resuspended in PBS. The MDA-MB-231 cell suspensions (5 × 10^4^ cells in 100 μl matrigel or 1 × 10^5^ cells in 100 μl PBS) were injected in the fourth mammary gland of each mouse. For MYLS22 treatment, a solution containing 10 mg/kg MYLS22 resuspended in corn oil was injected peritumorally from day 21 (when tumor size reached 50mm^3^) every second day. Thirty-six days after tumor cells injection, mice were euthanized, and their tumors harvested.

Tumor growth of MDA-MB-231 cells was assessed by measuring the length and width of each tumor every day. Tumor volume was calculated using the following formula:

$$\mathrm V=\mathrm l\;\ast\mathrm w^2\;\times\;0.5$$

Where V: volume; w: width; l: length.

#### Quantification and statistical analysis

##### Data representation and statistical analysis

Data are displayed as dot plots of at least 3 independent experiments. Plots include each datapoint, mean and SEM. In case of tumor growth curve, average ± SEM is shown. Normal distribution of populations at the 0.05 level was calculated using the Shapiro-Wilk normality test. Statistical significance was calculated by a one-way ANOVA with Tukey’s post-test using OriginPro (Microcal). All *P* values and n are reported in the figure legends. Results are considered significant when *p* < 0.05.

## Results

### Increased OPA1 levels associate with worse prognosis in breast cancer

**Fig. 1:**
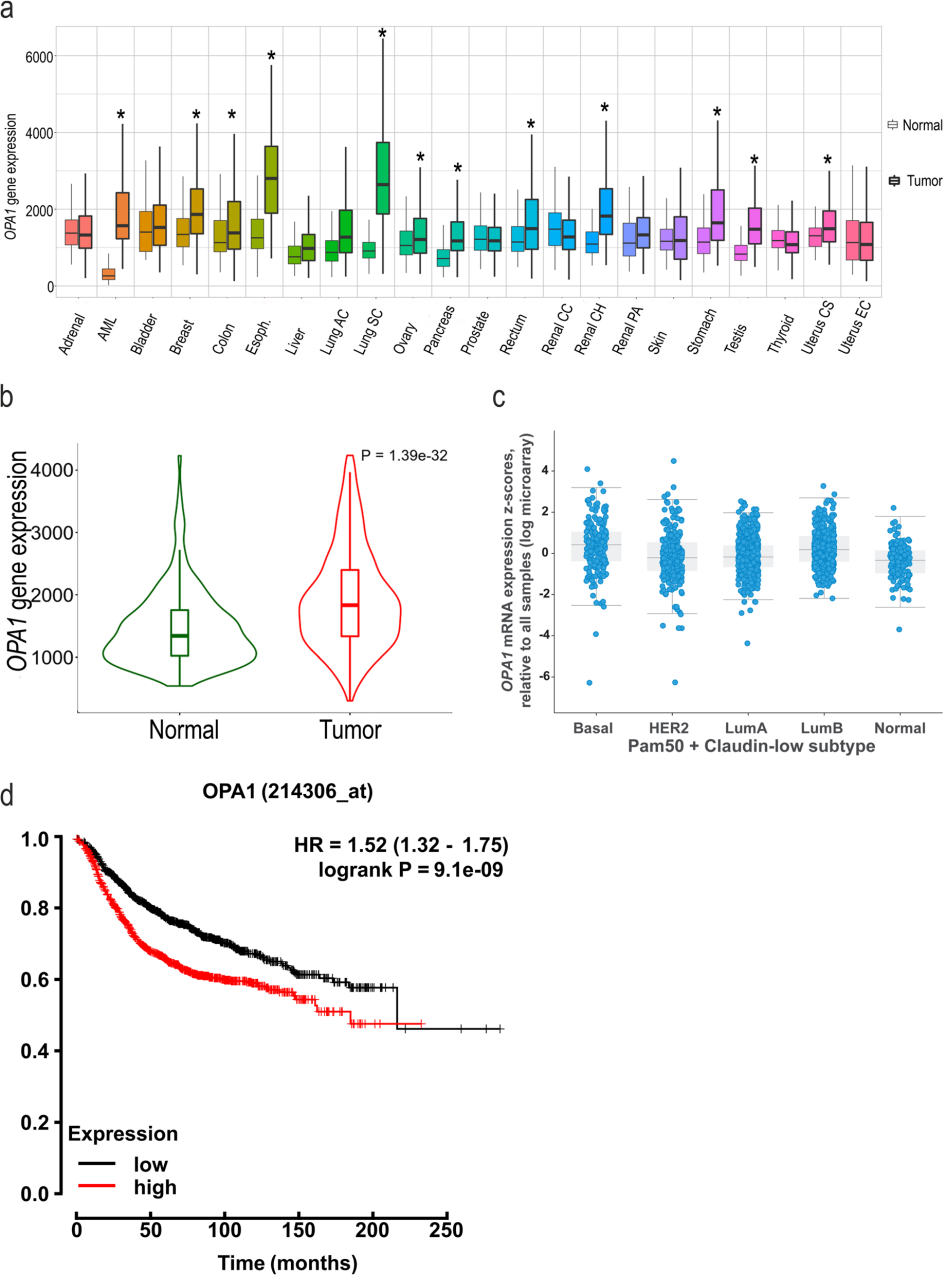
OPA1 is overexpressed in breast cancer tissue. **a** TNM plot of the OPA1 expression across all tissues in available normal and tumor RNA sequencing data (*n* = 15,648 normal, 40,442 tumor samples, *P < 0.05). Data are resorted from https://tnmplot.com. **b** Violin plot of OPA1 expression in breast invasive carcinoma from RNA sequencing data available. Data are resorted from https://tnmplot.com. **c** OPA1 mRNA expression in breast cancer type from METABRIC data using cbioportal (*n* = 1904 tumors). Tumors have been classified into five molecular intrinsic subtypes: Basal-like, HER2-enriched, Luminal A, Luminal B and Normal-like using the PAM 50 signature. **d** Kaplan-Meier survival curve for breast cancer patients stratified according to high (Q4) or low (Q1) *OPA1* levels. Data are resorted from Kaplan-Meier Plotter (http://kmplot.com). (Affymetrix id: 214306_at); *n* = 4934; Status: all. Follow up threshold: all

Unbiased approaches led to the identification of the key mitochondrial fission effector *DRP1* as a therapeutic liability of several cancer cell lines, including breast [33]. Because also mitochondrial fusion genes appear to be amplified in the same cancers, we wished to investigate whether levels of the three fusion mediators *OPA1*, *MFN1* and *MFN2* were upregulated across cancer types. To this end, we compared in the TNMplot data base their differential gene expression among healthy and tumor tissues [1]. Our analysis indicated that among the fusion genes, OPA1 was significantly overexpressed in AML, breast, colon, esophageal, lung, ovary, pancreatic, rectum, renal, stomach, testis, and uterus cancer tissues (Fig. 1a). MFNs were also differentially expressed among cancer tissues, *MFN1* increasing and *MFN2* decreasing (Suppl. Fig. 1a-b). We focused our attention on TNBC, and we delved into RNA sequencing databases (https://tnmplot.com/analysis/) to further evaluate whether also mRNA levels of mitochondrial fusion proteins were upregulated in breast cancer. We found that *OPA1* mRNA was indeed upregulated in breast cancer (Fig. 1b), without any difference across different breast cancer subtypes (basal, HER2, luminal A, luminal B and normal) (Fig. 1c). Conversely, mRNA levels of *MFN1* were slightly increased and of *MFN2* not affected (Suppl. Fig. 1c-d). Higher *OPA1* levels were strongly associated with worse breast cancer prognosis (Fig. 1d). We found also that MFN1 expression levels were also associated with worse prognosis, whereas MFN2 expression levels were not prognostic (Suppl. Fig. 1e-f). These analyses indicate that in breast cancer *OPA1* and *MFN1* are upregulated and prognosis is worse when levels of these two mitochondrial fusion genes, whereas *MFN2* levels are decreased, but not associated with a different prognosis.

### OPA1 is required for multiple cancer hallmarks of breast cancer cells

**Fig. 2:**
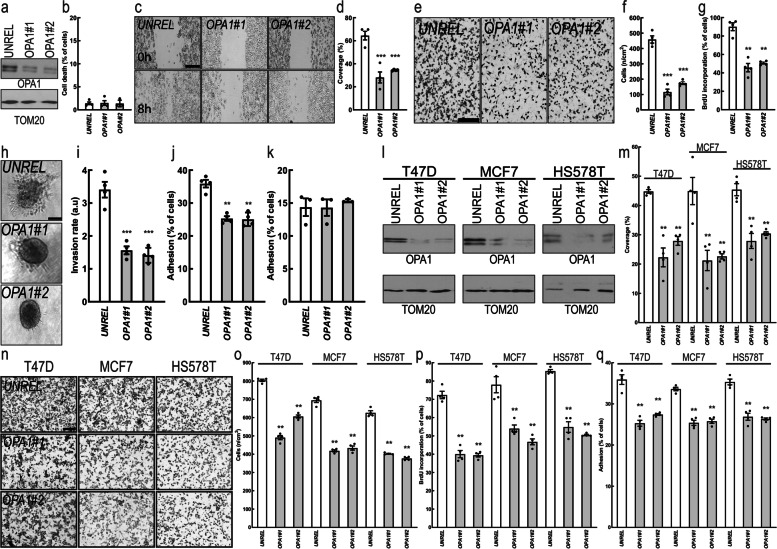
OPA1 is required for breast cancer cells hallmarks in vitro. **a** MDA-MB-231 cells were transfected with the indicated siRNA and lysed after 72 h. Equal amounts of proteins were separated by SDS-PAGE and immunoblotted with the indicated antibodies. **b** Quantification of apoptosis rate in MDA-MB-231 transfected with the indicated siRNA for 72 h determined by annexin V/propidium iodide label by flow cytometry. *n* = 4 independent experiments. **c** MDA-MB-231 cells were transfected with the indicated siRNA and after 72 h a scratch-wound assay was performed. Representative brightfield images were acquired at the indicated time points. Scale bar: 250 μm. **d** Quantification of cell migration after 6 h in *n* = 4 independent experiments as in **(b)**. ***: *p* < 0.0001. **e** Representative brightfield images of MDA-MB-231 cells transfected with the indicated siRNA in a Boyden chamber. Scale bar: 250 μm. **f** Quantification of cell migration in a Boyden chamber after 6 h in experiments as in **(d)**. *n* = 4 independent experiments. ***: *p* < 0.0001. **g** Quantification of MDA-MB-231 proliferation upon transfection with the indicated siRNA determined by BrdU incorporation. *n* = 4 independent experiments. **: *p* < 0.001. **h** Representative brightfield images of MDA-MB-231 spheroïds transfected with the indicated siRNA. Scale bar: 250 μm. **i** Quantification of cell invasion after 6 h in experiments as in **(h)**. *n* = 4 independent experiments. ***: *p* < 0.0001. **j** Quantification of cell adhesion on fibronectin for 1 h of MDA-MB-231 transfected with the indicated siRNA for 72 h. *n* = 4 independent experiments. ***: *p* < 0.001. **k** Quantification of cell adhesion on gelatin for 1 h of MDA-MB-231 cells transfected with the indicated siRNA for 72 h. *n* = 3 independent experiments. **l** Equal amounts of protein from breast cancer T47D, MCF7 and HS578T cells transfected for 72 h with the indicated siRNA were separated by SDS-PAGE and immunoblotted with the indicated antibodies. **m** Quantification of migration after 6 h in a scratch wound assay of T47D, MCF7 and HS578T cells transfected with the indicated siRNA for 72 h. *n* = 4 independent experiments. **: *p* < 0.001. **n** Representative brightfield images of T47D, MCF7 and HS578T cells transfected for 72 h with the indicated siRNA in a Boyden chamber 6 h after induction of migration. Scale bar: 250 μm. **o** Quantification of cell migration in experiments as in **(n)**. *n* = 4 independent experiments. **: *p* < 0.001. **p** Quantification of proliferation of T47D, MCF7 and HS578T transfected with the indicated siRNA determined by BrdU incorporation. *n* = 4 independent experiments. **: *p* < 0.001. **q** Quantification of cell adhesion on fibronectin for 1 h of T47D, MCF7 and HS578T cells transfected with the indicated siRNA for 72 h. *n* = 4 independent experiments. **: *p* < 0.001

**Fig. 3:**
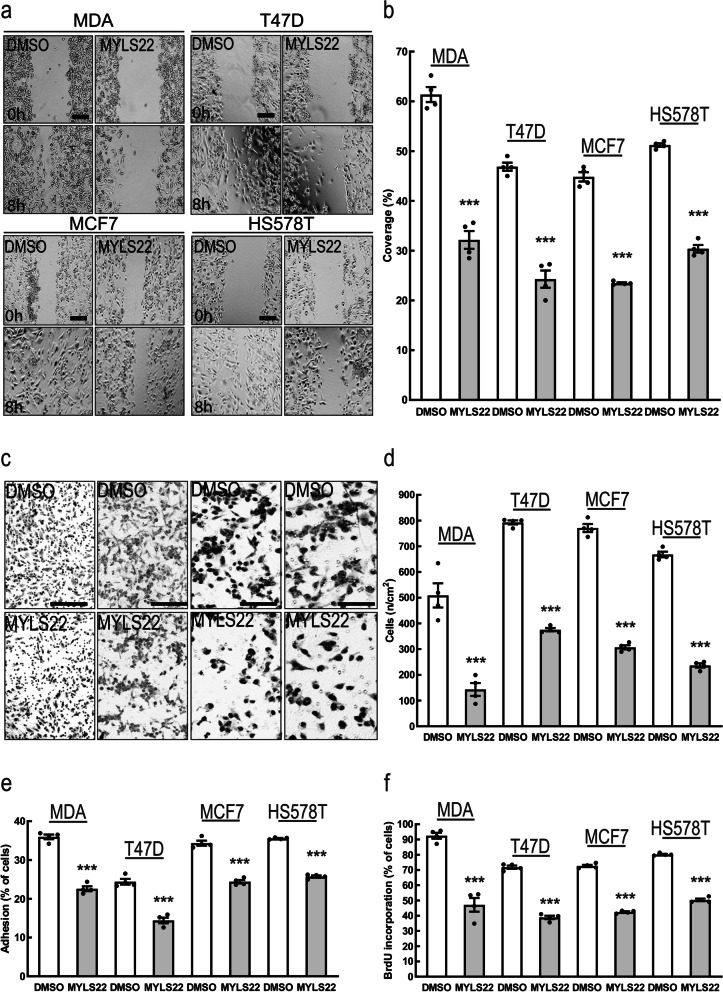
Pharmacological OPA1 Inhibition reduces breast cancer hallmarks in vitro. **a** Representative brightfield images acquired at the indicated time points of MDA-MB-231, T47D, MCF7 and HS578T cells incubated with MYLS22 (50 μM) or DMSO for 6 h in a scratch-wound assay. Scale bar: 250 μm. **b** Quantification of cell migration of MDA-MB-231, T47D, MCF7 and HS578T incubated with MYLS22 (50 μM) or DMSO, after 6 h in experiments as in **(a)**. *n* = 4 independent experiments. ***: *p* < 0.0001. **c** Representative brightfield images of MDA-MB-231,T47D, MCF7 and HS578T incubated with MYLS22 (50 μM) or DMSO for 6 h in a Boyden chamber. Scale bar: 200 μm. **d** Quantification of cell migration of MDA-MB-231,T47D, MCF7 and HS578T, incubated with MYLS22 (50 μM), after 6 h in experiments as in **(w)**. *n* = 4 independent experiments. ***: *p* < 0.0001. **e** Quantification of MYLS22- (50 μM) or DMSO-preincubated MDA-MB-231,T47D, MCF7 and HS578T adhesion on fibronectin for 1 h in presence of MYLS22 (50 μM) or DMSO. *n* = 4 independent experiments. ***: *p* < 0.0001. **f)** Quantification of MDA-MB-231,T47D, MCF7 and HS578Tproliferation incubated with MYLS22 (50 μM) for 24 h and determined by BrdU incorporation. *n* = 4 independent experiments. ***: *p* < 0.0001

Because levels of the mitochondrial fusion genes *MFNs* were not unequivocally associated like *OPA1* with worse breast cancer prognosis, we decided to concentrate our attention on the role of *OPA1* in breast cancer. We therefore measured the consequence of *OPA1* downregulation on key facets of cancer cell biology in MDA-MB-231, a TNBC cell line. While efficient *OPA1* downregulation by two different siRNAs (Fig. 2a) did not affect overall cell survival (Fig. 2b), it reduced MDA-MB-231 cells migration in a scratch wound (Fig. 2c-d) and in a Boyden chamber assay (Fig. 2e-f). Furthermore, *OPA1* silencing reduced cell proliferation (Fig. 2g), invasion (Fig. 2h-i) and adhesion on fibronectin, an extracellular matrix protein typical of the cancer niche (Fig. 2j) without affecting cellular adhesion on gelatin (Fig. 2k). Because siRNAs might have off target effects, we wished to verify our results in an MDA-MB-231 *OPA1* knockout cell line (MDA-MB-231 *OPA1*^−/−^) that we generated by CrispR/Cas9. We also reintroduced OPA1 in the MDA-MB-231 *OPA1*^−/−^ cells to generate MDA-MB-231 *OPA1*^−/−^:OPA1 cells. Immunoblotting confirmed the successful deletion and the reintroduction of OPA1 (Suppl. Fig. 2a). While survival of MDA-MB-231 *OPA1*^−/−^ cells was again comparable to that of their *OPA1*^+/+^ counterparts (Suppl. Fig. 2b), migration, proliferation, and adhesion were all reduced. Re-expression of OPA1 restored all the measured parameters, confirming that the phenotype was due to *OPA1* deletion (Suppl. Fig. 2c-f). To determine whether these effects were specific for *OPA1* deletion or generically caused by the perturbation of mitochondrial fusion, we silenced *MFN1* or *MFN2* in MDA-MB-231 cells (Suppl. Fig. 2g-h). Cell migration, proliferation and adhesion were not affected upon *MFN1* or *MFN2* downregulation (Suppl. Fig. 2i-p) even if, as expected, *MFN1* or *MFN2* silencing resulted in mitochondrial fragmentation (Suppl. Fig. 3a-c). Because our bioinformatic analysis did not reveal a difference in *OPA1* levels within breast cancer subtypes, we investigated whether its ablation in others breast cancer cell types caused the same phenotype observed in TNBC cells. We therefore silenced *OPA1* by siRNA in three other breast cancer cells that differ for histology, receptor and P53 status: T47D and MCF7, two Luminal-A ER+/PR+/HER2−/wild-type P53 cell lines; and HS578T, a Basal ER−/PR−/HER2−/wild-type P53 cell line (Fig. 2l). Silencing of *OPA1* inhibited migration, proliferation and adhesion also in these other breast cancer cells (Fig. 2m-q). Finally, we tested whether N-(1,5-dimethyl-3-oxo-2-phenyl-2,3-dihydro-1H-pyrazol-4-YL)-3-methyl-1-PH+ (MYLS22), the first in class safe and specific Opa1 inhibitor discovered in our laboratory, could recapitulate the effects of genetic OPA1 silencing on breast cancer cell phenotype. A non-toxic dose of MYLS22 inhibited breast cancer cells migration, proliferation and adhesion (Fig. 3a-f). These results indicate that genetic or pharmacological OPA1 inhibition reduces breast cancer cell proliferation, migration and invasiveness.

### OPA1 ablation impairs breast cancer growth in vivo

**Fig. 4:**
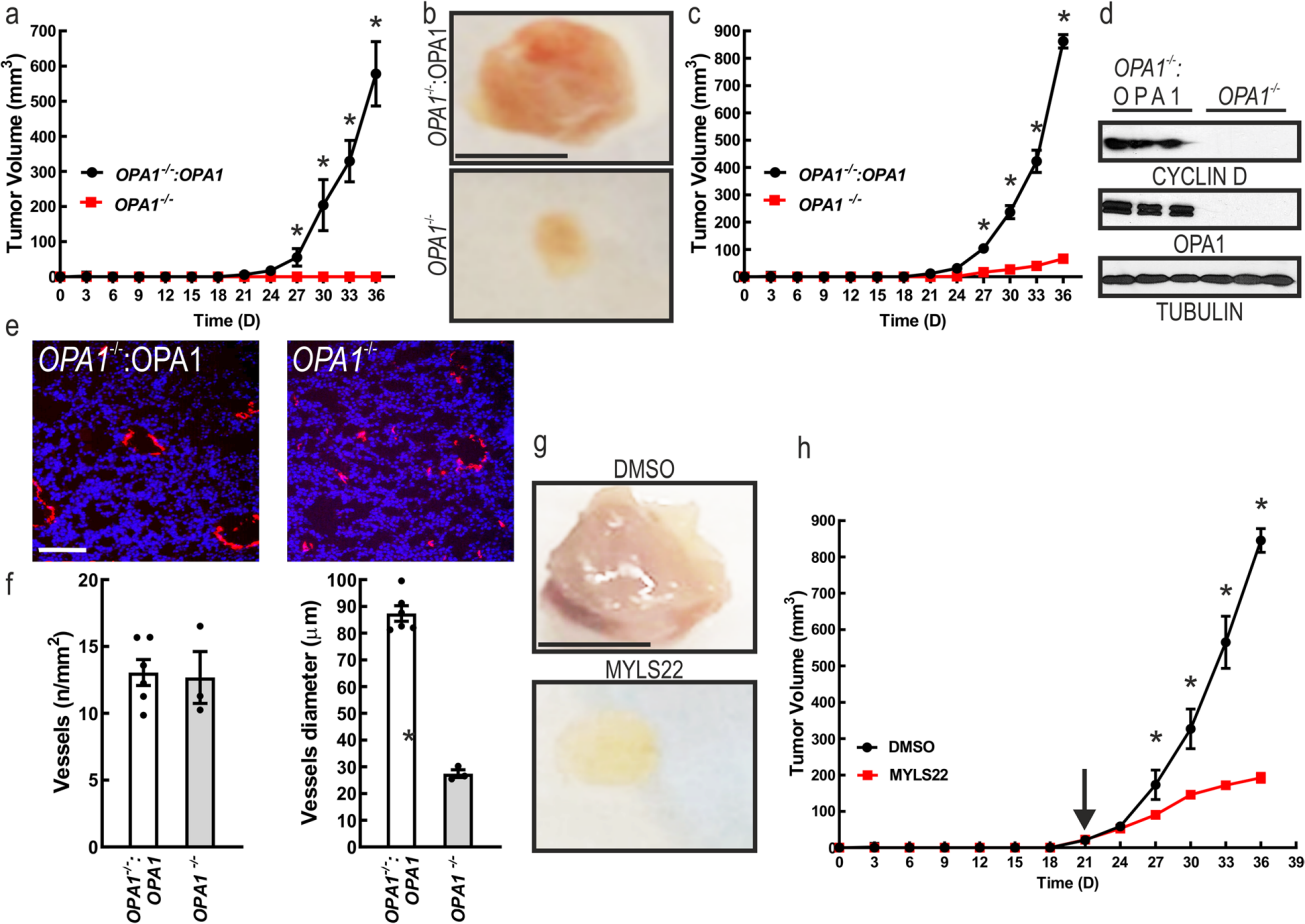
Genetic or pharmacological OPA1 inhibition curtails tumor growth. **a** Growth curves of orthotopically implanted 5 × 10^4^ MDA-MB-231 cells of the indicated genotype at day 0 in 6-week-old mice of the indicated genotype. *n* = 6. *: *p* < 0.05. **b** Representative photographs of breast adenocarcinoma removed after 35 days from orthotopically implants of 5 × 10^4^ MDA-MB-231 cells mixed with 50 μl of matrigel in 6-week-old mice of the indicated genotype. Scale bar: 0.5 cm. **c** Growth curves of orthotopically implanted 5 × 10^4^ MDA-MB-231 mixed with 50ul of Matrigel cells with the indicated genotype at day 0 in 6-week-old mice of the indicated genotype. *n* = 8 *Opa1*^*wt*^ and 8 *Opa1*^*Crispr*^ mice. *: *p* < 0.05. **d** Equal amounts of proteins from breast adenocarcinoma harvested from mice 35 days after implantation with the indicated genotype were separated by SDS-PAGE and immunoblotted with the indicated antibodies. **e** Representative CD31 immunofluorescence (red) showing blood vessels in MDA-MB-231 in experiments as in A. Scale bar: 100 μm. **f** Quantification of the number of tumor blood vessels in experiments as in **(d)**. **g** Quantification of tumor blood vessels diameter in experiments as in **(d)**. *:*P* < 0.05. **h** Representative photographs of breast adenocarcinomas removed after 35 days from orthotopically implants of 5 × 10^4^ MDA-MB-231 cells mixed with 50 μl Matrigel in 6-week-old mice treated as indicated. Scale bar: 0.5 cm. **i** Growth curves of orthotopically implanted 5 × 10^4^ MDA-MB-231 cells in 6-week-old mice treated as indicated. The arrow indicates the beginning of the treatment. *n* = 6. *: *p* < 0.05

We next investigated whether TNBC cells require OPA1 for growth in vivo. We implanted MDA-MB-231 *OPA1*^−/−^ and MDA-MB-231 *OPA1*^−/−^:OPA1 orthotopically in the mammary gland of 6-weeks old NOD-SCID mice. Surprisingly, we did not observe any engraftment (0/6 engrafted MDA-MB-231 *OPA1*^−/−^ vs. 6/6 MDA-MB-231 *OPA1*^−/−^:OPA1) and therefore growth of MDA-MB-231 *OPA1*^−/−^ cells, whereas the MDA-MB-231 *OPA1*^−/−^:OPA1 engrafted and grew (Fig. 4a). To circumvent the engraftment issue, we implanted MDA-MB-231 *OPA1*^−/−^ and MDA-MB-231 *OPA1*^−/−^:OPA1 cells in Matrigel. OPA1 deletion resulted in reduced engraftment (3/8 engrafted MDA-MB-231 *OPA1*^−/−^ vs. 8/8 MDA-MB-231 *OPA1*^−/−^:OPA1), but because 37.5% of the implants attached, we could compare tumor growth that was almost nil for the MDA-MB-231 *OPA1*^−/−^ compared to the MDA-MB-231 *OPA1*^−/−^:OPA1 grafts (Fig. 4b,c). At 36 days we explanted the tumors that displayed a degree of growth and measured levels of cyclin D, a proxy of cellular proliferation. Cyclin D was almost absent in the MDA-MB-231 *OPA1*^−/−^ explants, as opposed to the MDA-MB-231 *OPA1*^−/−^:OPA1 ones (Fig. 4d). In addition, when we visualized tumor vessels by immunostaining of breast cancer for the endothelial marker CD31, we noticed that vessels diameter but not number of vessels was reduced in the MDA-MB-231 *OPA1*^−/−^ tumors (Fig. 4e-g). These data suggested the possibility that acute OPA1 inhibition can counteract tumor growth. We therefore setup a proof of principle experiment to verify whether acute pharmacological OPA1 inhibition could curtail tumor growth. MDA-MB-231 were orthotopically implanted in the mammary gland and after 21 days (when tumors reached a 50mm^3^ volume) we delivered every second day by peritumoral injection 10 mg/kg of MYLS22. This regimen was sufficient to blunt the growth of MDA-MB-231 tumors (Fig. 4h-i). Thus, experiments of genetic as well as pharmacological inhibition nominate OPA1 as a targetable component of breast cancer in vivo.

### MicroRNA profiling identifies upregulated miRNAs upon OPA1 inhibition

**Fig. 5:**
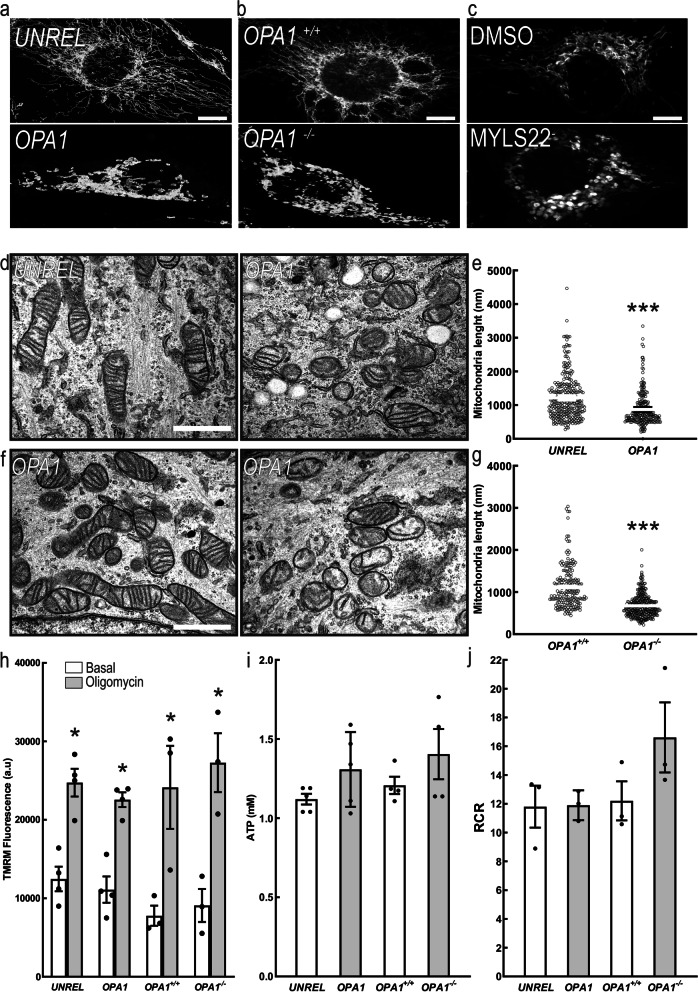
OPA1 ablation does not affect mitochondrial metabolism of breast cancer cells in vitro*.* Representative confocal images of mitochondrial morphology in MDA-MB-231 transfected with the indicated siRNA for 72 h and stained for TOM20. Scale bar: 30 μm. **a** Representative confocal images of mitochondrial morphology in MDA-MB-231 with the indicated genotype and stained for TOM20. Scale bar: 30 μm. **b** Representative confocal images of mitochondrial morphology in MDA-MB-231 treated 24 h with MYLS22 (50 μM) or DMSO and stained for TOM20. Scale bar: 30 μm. **c** Representative EM images of MDA-MB-231 transfected for 72 h with the indicated siRNA. Scale bar: 500 nm. **d** Quantification of mitochondrial length in experiments as in **(d)**. *n* = 240 mitochondria/condition from 3 independent experiments. *** *p* < 0.001. **e** Representative EM images of MDA-MB-231 with the indicated genotype. Scale bar: 500 nm. **f** Quantification of mitochondrial length in experiments as in **(f)**. *n* = 200 mitochondria/condition from 3 independent experiments. *** *p* < 0.001. **g** Quantitative analysis of mitochondrial TMRM fluorescence in MDA-MB-231 transfected with the indicated siRNA for 72 h; MDA-MB-231 *OPA1*^+/+^ and MDA-MB-231 *OPA1*^−/−^. Where indicated, cells were treated with the TMRM (0.5 nM), the ATP synthase inhibitor oligomycin (2 μg/mL) and the uncoupler FCCP (2 μM). Data represents mean ± SEM, *n* = 4. *: *p* < 0.05. **h** Total ATP levels were measured in MDA-MB-231 transfected with the indicated siRNA for 72 h; MDA-MB-231 *OPA1*^+/+^ and MDA-MB-231 *OPA1*^−/−^. *n* = 5 independent experiments. **i** Quantification of respiratory control ratio (RCR) calculated as State 3/ State 4 of MDA-MB-231 transfected with the indicated siRNA for 72 h; MDA-MB-231 *OPA1*^+/+^ and MDA-MB-231 *OPA1*^−/−^

**Fig. 6:**
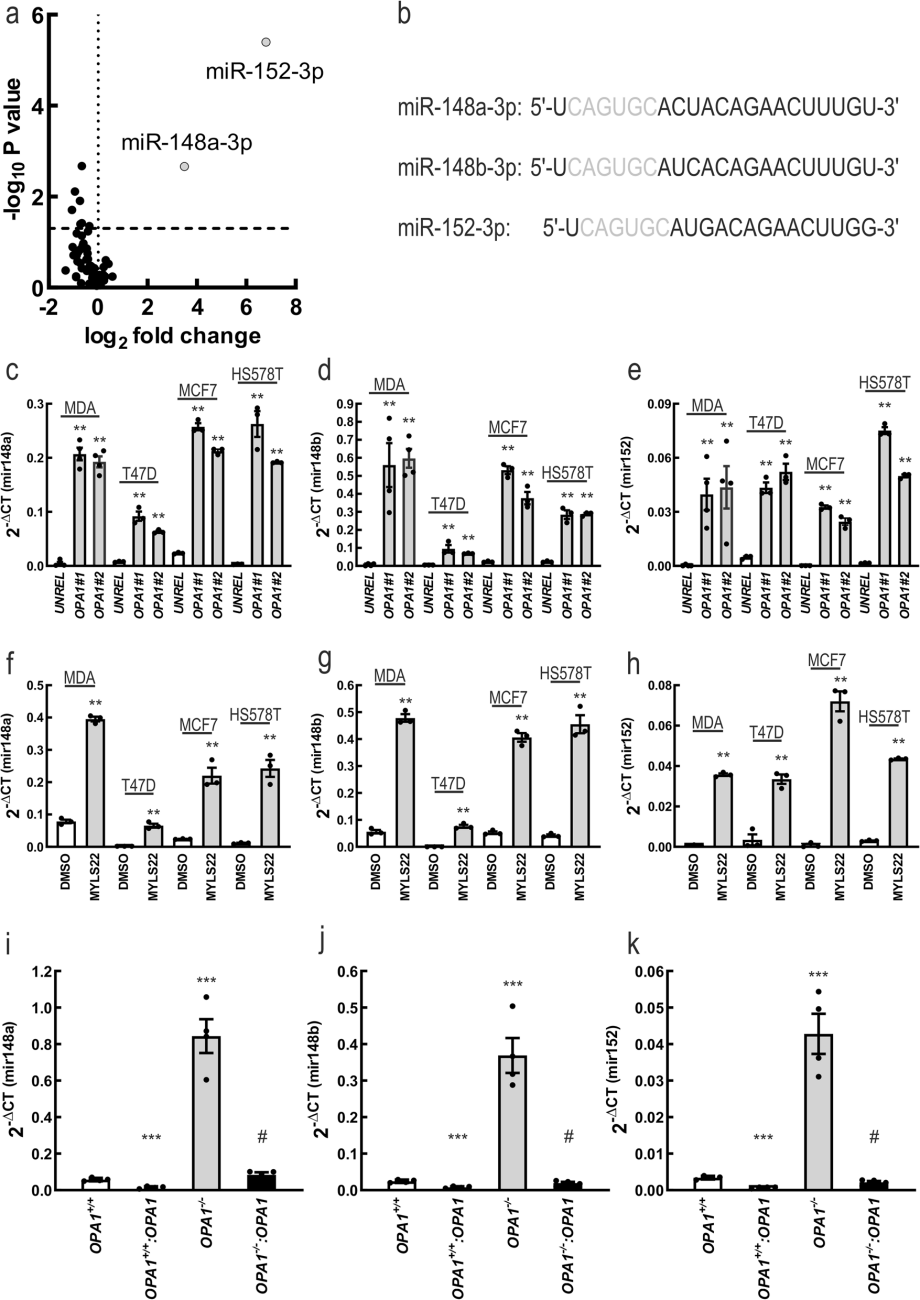
OPA1 ablation increases miR-148a, miR-148b and miR-152 expression level. **a** Volcano plot of differentially expressed miRNAs in MDA-MB-231 transfected for 72 h with siRNA against *OPA1* or a non-relevant sequence (*UNREL*). Blue dots correspond to miRNAs significantly up-regulated in Opa1-ablated MDA-MB-231. *n* = 4 independent experiments. **b** miR-148/152 family. Mature human and mouse sequences for miR-148a, miR-148b and miR-152. Seed sequences are colored in red. **c** 2^-Δct^ of *mir148a* levels determined by qRT-PCR in MDA-MB-231, T47D, MCF7, HS578T transfected as indicated for 72 h. *n* = 4 independent experiments. **: *p* < 0.001. **d** 2^-Δct^ of *mir148b* levels determined by qRT-PCR in MDA-MB-231, T47D, MCF7, HS578T transfected as indicated for 72 h. *n* = 4 independent experiments. **: *p* < 0.001. **e** 2^-Δct^ of *mir152* levels determined by qRT-PCR in MDA-MB-231, T47D, MCF7, HS578T transfected as indicated for 72 h. *n* = 4 independent experiments. **: *p* < 0.001. **f** 2^-Δct^ of *mir148a* levels determined by qRT-PCR in MDA-MB-231, T47D, MCF7, HS578T treated as indicated for 8 h. *n* = 4 independent experiments. **: *p* < 0.001. **g** 2^-Δct^ of *mir148b* levels determined by qRT-PCR in MDA-MB-231, T47D, MCF7, HS578T treated as indicated for 8 h. *n* = 4 independent experiments. **: *p* < 0.001. **h** 2^-Δct^ of *mir152* levels determined by qRT-PCR in MDA-MB-231, T47D, MCF7, HS578T treated as indicated for 8 h. *n* = 4 independent experiments. **: *p* < 0.001. **i** 2^-Δct^ of *mir148a* levels determined by qRT-PCR in MDA-MB-231 with the indicated genotype. *n* = 4 independent experiments. ***: *p* < 0.0001 #: *p* < 0.0001. **j** 2^-Δct^ of *mir148b* levels determined by qRT-PCR in MDA-MB-231 with the indicated genotype. *n* = 4 independent experiments. ***: *p* < 0.0001 #: *p* < 0.0001. **k** 2^-Δct^ of *mir152* levels determined by qRT-PCR in MDA-MB-231 with the indicated genotype. *n* = 4 independent experiments. ***: *p* < 0.0001 #: *p* < 0.0001

We next wished to understand how OPA1 inhibition could influence these multiple aspects of breast cancer cell biology. A likely explanation was that *OPA1* silencing led to mitochondrial dysfunction i.e., reduced mitochondrial respiration and membrane potential. However, while genetic or pharmacological OPA1 inhibition induced the expected mitochondrial fragmentation in MDA-MB231 cells (Fig. 5a-g), mitochondrial membrane potential, ATP level and mitochondrial respiration were not affected in *OPA1*-silenced and *OPA1*-ablated MDA-MB-231 (Fig. 5 h-j). Another potential explanation for the pleiotropic effects observed upon *OPA1* deletion in breast cancer cells was the potential regulation of miRNAs that can control multiple cellular pathways by seeding on a variety of different 3’UTRs. In the case of breast cancer, several miRNAs have been identified to regulate the same biological effects caused by OPA1 deletion [34–36]. We therefore performed a miRNA profiling by using miRCURY LNA Cancer Focus PCR Panel (breast cancer panel) to investigate if *OPA1* deletion affected miRNAs expression. The comparison of PCR array data obtained from four replicate analyses of control and *OPA1*-ablated MDA-MB-231 cells indicated that only 6 miRNAs out of a total of 84 were differentially expressed [fold change (FC), threshold 0.5; FDR = 0.05, Fig. 6a]. Interestingly, miR-148a-3p and miR-152 were the top two upregulated miRNAs. These two miRNAs belong to the same miRNA148/152 family that consists of three members, miR-148a, miR-148b and miR-152 [2]. Mature members of the mir-148/152 family are 21–22 nucleotides in length, with the same seed sequence of 6–7 nucleotides (red in Fig. 6b). We confirmed by quantitative PCR (qPCR) the increase in miRNAs 148a, 148b and 152 levels upon *OPA1* downregulation or pharmacological inhibition in all the breast cancer cell types tested (Fig. 6c-h). Levels of miRNAs 148a, 148b and 152 levels were also increased in MDA-MB-231 *OPA1*^−/−^ cells, whereas they were normalized in MDA-MB-231 *OPA1*^−/−^:OPA1 cells. When we overexpressed OPA1 in MDA-MB-231 *OPA1*^+/+^ cells (MDA-MB-231 *OPA1*^+/+^:OPA1), levels of these miRNAs were further decreased (Fig. 6i-k). To determine whether miRNAs148a, 148b and 152 were specifically induced upon *Opa1* deletion, we also measured their levels following *MFN1* or *MFN2* silencing. Interestingly, levels of miR-148a, 148b and 152 did not increase upon silencing of the MFNs, but we observed a decrease in miR-148a levels upon *MFN1* downregulation and of miR-148b when we silenced *MFN2* (Suppl. Fig. 4a-c). Finally, overexpression of the two MFNs alone or in combination did not affect levels of these miRNAs (Suppl. Fig. 4d-f). Altogether, these results indicate that levels of miRNAs of the 148/152 family inversely correlate with OPA1, but not MFNs levels.

### miRNAs of the 148/152 family mediate the effects of OPA1 downregulation in MDA-MB231 cells

**Fig. 7:**
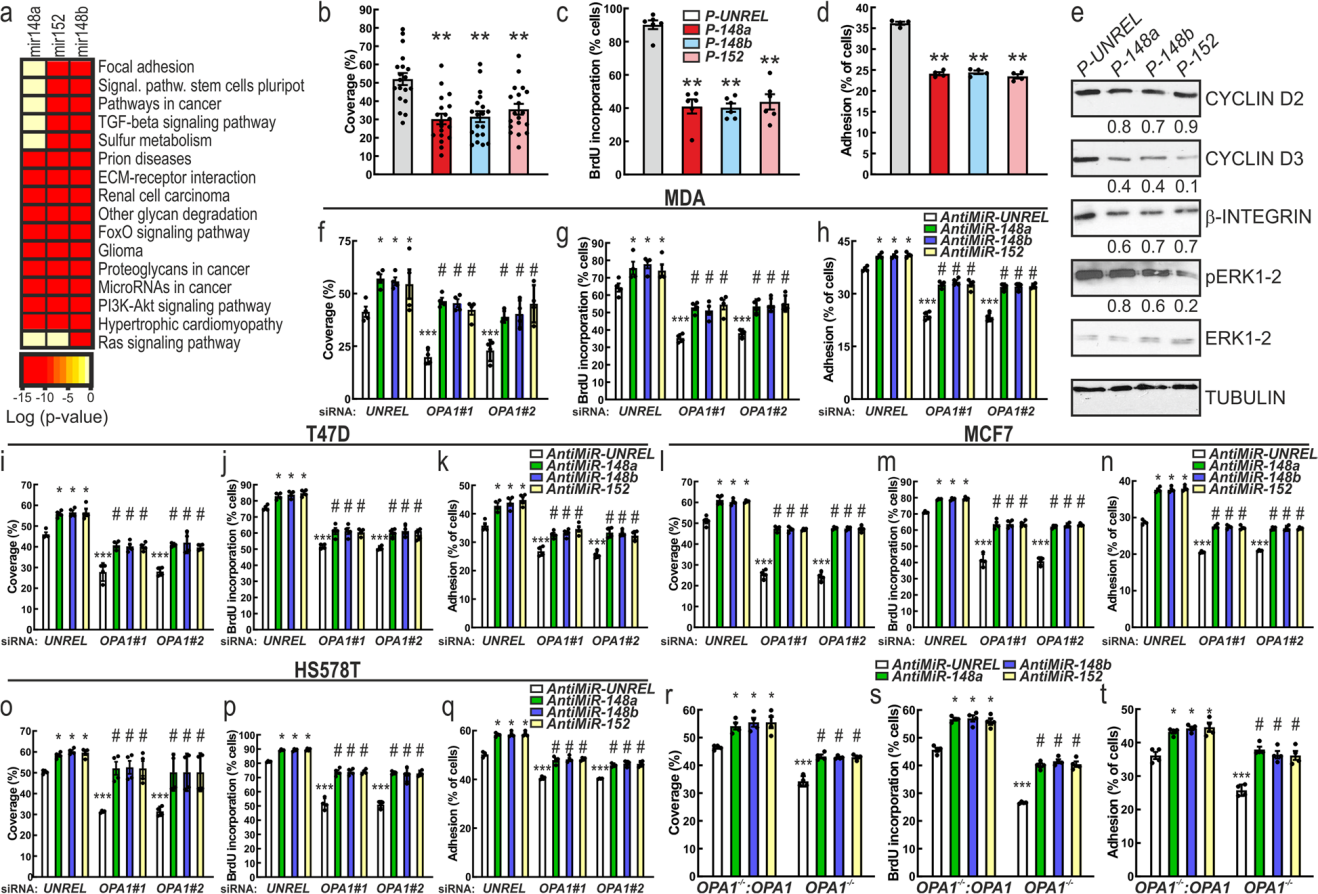
OPA1 ablation inhibits breast cancer cells hallmarks by increasing miRNA 148a, 148b and 152 expression. **a** Heat map of the major pathways dysregulated by the change of the miRNA-148a, 148b and 152. Data are resorted from http://snf-515788.vm.okeanos.grnet.gr/#mirnas=hsa-miR-148a-3p;hsa-miR-148b-3p;hsa-miR-152-3p&methods=microT-CDS;microT-CDS;microT-CDS&selection=2. **b** Quantification of cell migration after 6 h of MDA-MB-231 transfected as indicated for 24 h. *n* = 19 independent experiments. **: *p* < 0.001. P-: miRNA mimic. **c** Quantification of proliferation of MDA-MB-231 transfected as indicated for 24 h determined by BrdU incorporation. *n* = 4 independent experiments. **: *p* < 0.001. P-: miRNA mimic. **d** Quantification of cell adhesion on fibronectin of MDA-MB-231 transfected as indicated for 24 h. *n* = 4 independent experiments. **: *p* < 0.001. P-: miRNA mimic. **e** Equal amounts of protein from breast cancer cells MDA-MB-231 transfected 24 h as indicated were separated by SDS-PAGE and immunoblotted with the indicated antibodies. Numbers above each panel represent densitometric quantification. P-: miRNA mimic. **f-h** Quantification of cell migration (**f**), proliferation (**g**) and adhesion (**i**) of MDA-MB-231 (transfected with the indicated siRNA for 72 h and indicated anti-miRNA for 24 h. *n* = 4 independent experiments. *: *p* < 0.05, ***: *p* < 0.0001 and #: *p* < 0.05. A-: Anti miRNA. **i-k** Quantification of migration (**i**), proliferation (**j**) and adhesion (**k**) of T47D transfected with the indicated siRNA for 72 h and indicated anti-miRNA for 24 h and determined by BrdU incorporation. *n* = 4 independent experiments. *: *p* < 0.05, ***: *p* < 0.0001 and #: *p* < 0.05. A-: Anti miRNA. **l-n** Quantification of cell migration (**l**), proliferation (**m**) and adhesion (**n**) of MCF7 transfected with the indicated siRNA for 72 h and indicated anti-miRNA for 24 h. *n* = 4 independent experiments. *: *p* < 0.05, ***: *p* < 0.0001 and #: *p* < 0.05. A-: Anti miRNA. o-q Quantification of cell migration (**o**), proliferation (**p**) and adhesion (**q**) of HS578T transfected with the indicated siRNA for 72h and indicated anti-miRNA for 24h. n=4 independent experiments. *: p < 0.05, ***: p < 0.0001 and #: p < 0.05. A-: Anti miRNA.  **r** Quantification of cell migration after 6 h of MDA-MB-231 with the indicated genotype and transfected as indicated for 24 h. *n* = 4 independent experiments. *: *p* < 0.05, ***: *p* < 0.0001 and #: *p* < 0.05. A-: Anti miRNA. **s** Quantification of proliferation of MDA-MB-231 with the indicated genotype and transfected as indicated for 24 h and determined by BrdU incorporation *n* = 4 independent experiments. *: *p* < 0.05, ***: *p* < 0.0001 and #: *p* < 0.05. A-: Anti miRNA. **t** Quantification of cell adhesion on fibronectin of MDA-MB-231 with the indicated genotype and transfected as indicated for 24 h. *n* = 4 independent experiments. *: *p* < 0.05, ***: *p* < 0.0001 and #: *p* < 0.05. A-: Anti miRNA

To ascertain whether miR-148a, 148b and 152 overexpression could explain the phenotype by *OPA1* downregulation in MDA-MB-231 cells, we first analyzed by miRpaths (microT-CDS) the signaling pathways and cellular functions that might be perturbed by changes in expression levels of these three miRNAs. Interestingly, miR-148a, 148b and 152 had the potential to regulate the RAS and PI3K/AKT signaling pathways. Additionally, miR-148a, 148b and 152 could also control extracellular matrix (ECM)-receptor interaction, focal adhesion and glycan degradation (Fig. 7a). Therefore, the observed increase of miR-148a, 148b and 152 expression levels following *OPA1* downregulation could at least partially explain the observed phenotype. To verify this possibility, we first analyzed if overexpression of miR-148a, miR-148b or miR-152 in MDA-MB-231 cells phenocopied *OPA1* silencing. When we transfected MDA-MB-231 cells with miR148a, 148b or 152 mimics, levels of miR-148a, miR-148b and miR-152 increased (Suppl. Fig. 5a-c). Overexpression of these miRNAs reduced MDA-MB-231 cells migration, proliferation, and adhesion (Fig. 7b-d). In line with these results, overexpression of these miRNA reduced *CYCLIN D2* and *D3*, *INTEGRIN-β* mRNA and protein levels and therefore decreased ERK phosphorylation (Fig. 7e and Suppl. Fig. 5d-f). Conversely, delivery of anti-miRNA-148a, 148b or 152 reduced the expression of these miRNAs (Suppl. Fig. 5 g-i) and increased breast cancer cells migration, proliferation and adhesion (Suppl. Fig. 5j-l). Additionally, downregulation of these miRNA increased *CYCLIN D2* and *D3*, *INTEGRIN-β* mRNA and protein levels and therefore increased ERK phosphorylation (Suppl. Fig. 5 m-p). To formally verify whether the phenotype observed in MDA-MB-231 cells upon *Opa1* silencing was mediated by an increase in 148/152 family miRNAs, we performed an epistatic analysis and overexpressed miR-148a, miR-148b or miR-152 in *OPA1*-silenced cells. Silencing of miR-148a, miR-148b or miR-152 rescued migration, proliferation and adhesion of breast cancer cells where we had silenced OPA1 (Fig. 7f-t). Conversely, we did not observe any additive effect of *OPA1* silencing and simultaneous miRNAs overexpression on MDA-MB-231 migration, proliferation and adhesion (Suppl. Fig. 6a-c). These results establish the role of miRNAs of the 148/152 family as mediators of *OPA1* silencing in breast cancer cells.

## Discussion

OPA1 levels are higher in several tumors including AML, breast, colon, esophageal, lung, ovary, pancreatic, rectum, renal, stomach, testis and uterine cancer, and they positively correlate with resistance to conventional chemotherapeutics [25–29, 37], as predicted given the role of OPA1 as apoptosis inhibitor [24, 30, 38] and angiogenesis initiator [39]. However, whether cancer cells are addicted to OPA1, and its pharmacological or genetic inhibition can curtail tumor growth awaits formal testing. Our study shows that *OPA1* ablation or inhibition with MYLS22, a specific, nontoxic small molecule inhibitor identified in our laboratory [39] decreases breast cancer cell migration, proliferation, adhesion and invasion. The pleiotropic effects of Opa1 inhibition are not a consequence of mitochondrial dysfunction, but mediated by the upregulation of specific miRNAs, explaining how changes in multiple complex cellular processes can be orchestrated by the inhibition of a single mitochondrial protein.

OPA1 function extends beyond the regulation of mitochondrial fusion. The effects of OPA1 ablation on breast cancer progression could therefore be OPA1 specific, or a consequence of inhibition of mitochondrial fusion per se. Even though MFN1 expression levels in breast cancer also correlate with worse prognosis, our data indicate that *MFN1* or *MFN2* deletion does not affect breast cancer cells migration, proliferation, or adhesion. This picture is similar to that observed in the case of apoptosis, angiogenesis, or regulation of mitochondrial metabolism, where the effect of OPA1 is not phenocopied by the MFNs [23, 24, 39, 40], and these results are important in the context of the ongoing efforts to understand the role of mitochondrial morphology vs. that of each of these individual proteins with pleiotropic functions beyond the control of mitochondrial shape in breast cancer. Indeed, the mitochondrial fission protein Drp1 also increases breast cancer sensitivity towards apoptosis or chemotherapy. Similarly, early studies indicated that OPA1 downregulation sensitizes different cells to apoptotic inducers [41]. This is not surprising given the role of OPA1 as a master regulator of cristae remodeling and cytochrome c release. However, *OPA1* deletion in breast cancer cells is not associated with mitochondrial dysfunction and cell death. This is in line with what observed in other cell lines and tissues where OPA1 deletion does not cause mitochondrial dysfunction [23, 39]. Conversely, we posit that the effect of OPA1 on breast cancer is specific and could be mediated by its ability to control the transcription of miRNAs. The two upregulated miRNAs, 148a and 152, belong to the miRNA148/152 family consisting also of miRNA148b [42]. This miRNA family is known to function as tumor suppressor (anti-oncomiRs). Indeed, their downregulation occurs in a number of cancer cell lines and tumor tissues including gastrointestinal cancer, endometrial cancer, ovarian cancer, hepatocellular carcinoma, colorectal cancer and breast cancer [43–46]. Often, this downregulation is caused by hypermethylation of their promoter region by the DNA methyltransferase 1 (DNMT1) that is overexpressed in several cancer [47–50]. Overexpression of miR-148a, miR-148b or miR-152 in breast cancer cell lines reduces migration, proliferation, colony formation and their ability to induce angiogenesis [43, 44, 51], suggesting that the miR148/152 family can act as tumor suppressors in breast cancer [43, 44, 52]. Our formal epistatic analysis led to the conclusion that OPA1 ablation requires upregulation of these miRNAs to reduce breast cancer cells growth. How changes in OPA1 levels affect transcription of these miRNAs remains to be understood. One possibility is that *OPA1* deletion results in changes that reduce DNMT1 activity. Interestingly, increased Ca^2+^ levels convert DNMT1 into a demethylase [53] and *OPA1* deletion increases intracellular Ca^2+^ levels [39]. Alternatively, *OPA1* deletion might also lead to accumulation of alpha-ketoglutarate, a key factor to activate the ten-eleven translocation (TET) family of epigenetic modifiers that demethylate DNA, albeit unbiased metabolomic on Opa1-deficient fibroblasts reported a reduction in alpha-ketoglutarate levels [54].

The connection between OPA1 inhibition and levels of miRNAs found here might sound surprising. A handful of studies identified miRNAs as modulators of mitochondrial function, whereas the opposite i.e., that mitochondria can modulate miRNA levels is less explored. However, mitochondria-derived oncometabolites have been shown to regulate expression of miRNAs. For example, fumarate that accumulates in FH deficient renal cells drives their epithelial-mesenchymal transition by upregulating miR-200 [55].

## Conclusions

In summary, we demonstrate that OPA1 is highly expressed in breast cancer where its levels correlate with worse prognosis. By using a multipronged approach, we substantiate that OPA1 inhibition curtails TNBC growth in vitro and in vivo, by counteracting tumor growth, invasiveness, and neovascularization. Surprisingly, our data identify a role for OPA1 in the regulation of the expression of miRNAs of the 148/152 family that are epistatic to OPA1 in the modulation of breast cancer hallmarks. We believe that our study nominates OPA1 as a potential target in cancer therapy.

## Supplementary Information

**Additional file 1.**

## Data Availability

Further information and requests for resources and reagents should be directed to and will be fulfilled by the Lead Contact, LS (luca.scorrano@unipd.it). Cell line generated in this study (*Opa1*^*Crispr*^ MDA-MB-231) is available from the lead contact upon request.

## Abbreviations

AML: Acute myeloid leukemia
AR: Androgen Receptor
B2M: Beta-2 microglobulin
BL1: Basal-like 1
BL2: Basal-like 2
BLIA: Basal-like immune-activated
BLIS: Basal-like immunosuppressed
DNMT1: DNA methyltransferase 1
DRP1: Dynamin-related protein 1
ECM: Extracellular matrix
ER: Estrogen receptor
Fis1: Fission 1
IM: Immunomodulatory
IMM: Inner mitochondrial membrane
LAR: Luminal androgen receptor
M: Mesenchymal
MFF: Mitochondrial fission factor
MFN: Mitofusin
Mid49: Mitochondrial division 49
Mid51: Mitochondrial division 51
miRNA: Micro-RNA
MSL: Mesenchymal stem-like
OMM: Outer mitochondrial membrane
OPA1: Optic Atrophy 1
OXPHOS: Oxidative Phosphorylation
PARP: Poly-ADP ribose polymerase
PPIA: Cyclophilin-A
PR: Progesterone receptor
qPCR: Quantitative PCR
TNBC: Triple negative breast cancer
