# Supplementary material for: Inhibition of the mitochondrial protein Opa1 curtails breast cancer growth
Source: J Exp Clin Cancer Res. 2022 Mar 12;41:95. doi: 10.1186/s13046-022-02304-6 (PMC8917763; doi:10.1186/s13046-022-02304-6)
Supplement: Supplementary file 1 — Additional file 1. [file 13046_2022_2304_MOESM1_ESM.pdf]

Inhibition of the mitochondrial protein Opa1 curtails breast cancer growth.

Margherita Zamberlan, Amandine Boeckx et al.

Supplementary material

Supplementary Methods

KEY RESOURCES TABLE

REAGENT or RESOURCE	SOURCE	IDENTIFIER
**Antibodies**		
Rabbit anti-cyclinD2	Cell Signalling	3741
Mouse anti-Opa1	BD-Biosciences	612607
rabbit anti-GRP75	Santa Cruz Biotechnology	sc-13967
Mouse anti-Tom20	Santa Cruz Biotechnology	
Mouse anti-cyclinD3	Cell signaling	2936
Rabbit anti-Tom20	Invitrogen	
Rat anti mouse CD31	BD Pharmingen	550274
Anti mitofusin 1	NeuroMabs	75162
Anti mitofusin 2	ABM	Y401293
Rabbit anti-ERK1/2-phospho, p44/42 MAPK Phospho	Cell Signaling Technology	9101
Rabbit anti-ERK1/2, p44/42 MAPK	Cell Signaling Technology	4695
Rabbit anti-AKT pan (11EZ)	Cell Signaling Technology	4685
Rabbit anti-Phospho-Akt (Ser473) Antibody	Cell Signaling Technology	9271
Alexa Fluor 488 goat anti mouse IgG	Life Technologies	A11029
Alexa Fluor 488 goat anti rabbit IgG	Life Technologies	A11070
Alexa Fluor 488 Chicken anti-Rat IgG	Life Technologies	A21470
Alexa Fluor 594 Donkey anti-Rabbit IgG	Thermo fisher	A10042
Alexa Fluor 568 Goat anti-Rat IgG	Thermo fisher	A-11077
Alexa Fluor 568 Goat anti-Mouse IgG	Thermo fisher	A-11019
**Chemicals, Peptides, and Recombinant Proteins**		
BAPTA, AM	Thermo Fisher Scientific	B6769
Fura-2, AM	Life Technologies	F1221
Rotenone	Sigma-Aldrich	R8875-1G
FCCP	Sigma-Aldrich	C2920
Antimycin A	Sigma-Aldrich	A8674
Oligomycin	Sigma-Aldrich	O4876
TMRM	Invitrogen	T668

MYLS22	Enamine	EN300-333633
Thapsigargin	Sigma-Aldrich	T903
Trypan Blue Solution, 0.4%	Thermo Fisher Scientific	15250061
Matrigel matrix 3D	VWR	7341100
Dharmafect 4	Dharmacon	T-2004-03
**Critical Commercial Assays**		
Takyon ROX SYBR MasterMix blue dTTP	Eurogentec	UF-RS MT-B0701
Poly5A Tailing and reverse transcription of miRNA	Biolabs	
iScript cDNA Synthesis Kit	Biorad	1708890
E. coli Poly(A) Polymerase	Biolabs	M0276S
Deoxynucleotide (dNTP) Solution Mix	Biolabs	N0447L
M-MuLV Reverse Transcriptase	Biolabs	M0253S
Breast Cancer Focus microRNA PCR Panel, 96-well	Qiagen	
ATPlite	Perkin Elmer	6016943
BrdU Chemiluminescence Kit	Roche	11647229001
RNeasy Mini Kit	Qiagen	74104
Experion RNA StdSens Analysis kit	Bio-Rad	7007103
Pierce protein BCA assay	Thermo Fisher	23225
Annexin V-FITC Apoptosis Detection Kit	eBioscience	BMS500FI/300
**Experimental Models: Cell Lines**		
T47D	Dr. Gilles, U. of Liège	
MDA MB231	ATCC	ATCC-HTB26
MCF7	Dr. Gilles, U. of Liège	
HS598T	Dr. Gilles, U. of Liège	
**Experimental Models: Organisms/Strains**		
NOD SCID mice	U of Liège int. breeding	N/A
**Oligonucleotides**		
siRNA		
OPA1 siRNA 1	Invitrogen	144409
OPA1 siRNA 2	Invitrogen	36409
Unrel	Ambion	AM4635
MFN1 siRNA 1 5'-GGC GAU UAC UGC AAU CUU U-3',	DeBrito&Scorrano, 2008	N/A
MFN1 siRNA2 5'-CCA GAU GAA CCU UUU AAC A-3'	DeBrito&Scorrano, 2008	N/A

MFN2 siRNA1 5'-GGA GAG GGC CUU CAA GCG C-3'	DeBrito&Scorrano, 2008	N/A
MFN2 siRNA2 5'-GAG ACA CAU GGC UGA GGU G-3'	DeBrito&Scorrano, 2008	N/A
**Primers**		
Cyclin D1 oligonucleotide for CAATGACCCCGCACGATTTC	Eurogentec	
Cyclin D1 oligonucleotide rev CATGGAGGGCGGATTGGAA	Eurogentec	
Cyclin D2 oligonucleotide for CTCGAGGGATGCCAGTTGGGCC	Eurogentec	
Cyclin D2 oligonucleotide rev GCGGCCGCCAAAAGCGTGAATCATTGCC	Eurogentec	
Cyclin D3 oligonucleotide for TACCCGCCATCCATGATCG	Eurogentec	
Cyclin D3 oligonucleotide rev AGGCAGTCCACTTCAGTGC	Eurogentec	
B2M oligonucleotide for GAGTATGCCTGCCGTGTG	Eurogentec	
B2M oligonucleotide rev AATCCAAATGCGGCATCT	Eurogentec	
PPIA oligonucleotide for CCAACACAAATGGTTCCCAGT	Eurogentec	
PPIA oligonucleotide rev CCATGGCCTCCACAATATTCA	Eurogentec	
OPA1 oligonucleotide for AGCCTCGCAGGAATTTTTGG	Eurogentec	
OPA1 oligonucleotide rev AGCCGATCCTAGTATGAGATAGC	Eurogentec	
MFN1 oligonucleotide for ATGACCTGGTGTTAGTAGACAGT	Eurogentec	
MFN1 oligonucleotide rev AGACATCAGCATCTAGGCAAAAC	Eurogentec	
MFN2 oligonucleotide for CACATGGAGCGTTGTACCAG	Eurogentec	
MFN2 oligonucleotide rev TTGAGCACCTCCTTAGCAGAC	Eurogentec	
INTB8 oligonucleotide for CGT GAC TTT CGT CTT GGA TTT GG	Eurogentec	
INTB8 oligonucleotide rev TCC TTT CGG GGT GGA TGC TAA	Eurogentec	
COL4A oligonucleotide for GGG ATG CTG TTG AAA GGT GAA	Eurogentec	

COL4A oligonucleotide rev GGT GGT CCG GTA AAT CCT GG	Eurogentec	
SNORD44 gcaaatgctgactgaacatgaa	Eurogentec	
SNORD48 ctctgagtgtgtcgctgatgc	Eurogentec	
**Software and Algorithms**		
Prism	GraphPad	
Photoshop	Adobe	
Image J V1.38	NIH	imagej.nih.gov/ij/
Geneglobe	QiaGEN	
**Other**		
Gelatin from bovine skin	SIgma aldrich	G9391
DMEM	Gibco	31885049
Penicillin-streptomycin (5000U/ml)	Invitrogen	15070-063
cOmplete™, Mini EDTA-free Protease Inhibitor Cocktail	Roche	4693159001
PhosStop Phosphatase Inhibitor Cocktail Tablets	Roche	04906845001
Amersham Hybond 0.45um PVDF	Thermo Fisher Scientific	15407374
ExpressPlus PAGE Gel, 10x8, 8% 12 wells	GenScript	M00812
ExpressPlus PAGE Gel, 10x8, 4-12% 10 wells	GenScript	M41210
Trypsin-EDTA (0.25%)	Thermo Fisher	25200056
Bovine Serum Albumin	Sigma Aldrich	A8806
Ketamidor 100mg/mL (Ketamine)	Ecuphar	3026770
Proxylaz 20mg/mL (Xylazine)	Prodivet pharmaceuticals	Nc

Supplementary Figures

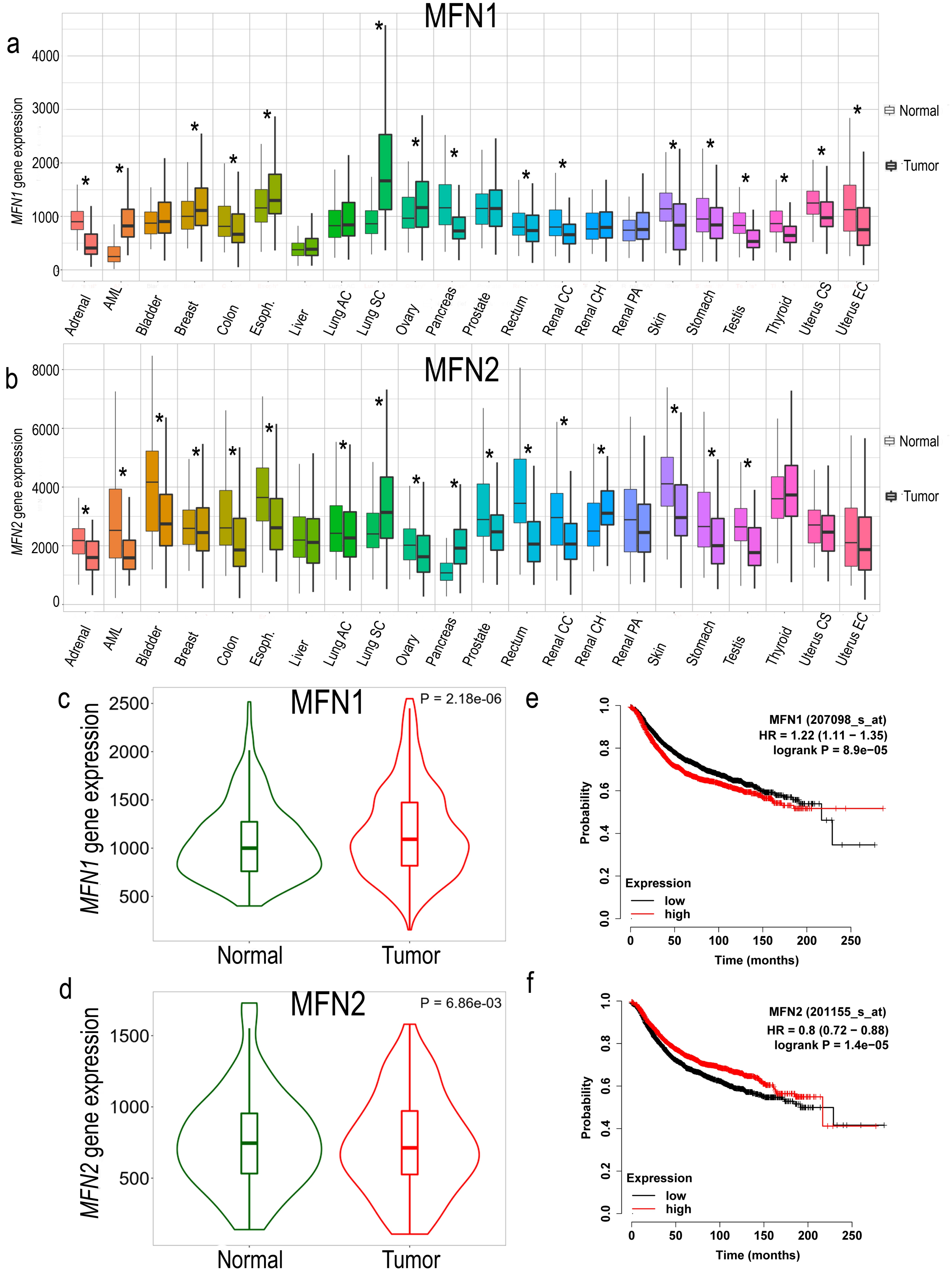
a
MFN1
MFN1 gene expression
4000
3000
2000
1000
0
Normal
Tumor
Adrenal
AML
Bladder
Breast
Colon
Esoph.
Liver
Lung AC
Lung SC
Ovary
Pancreas
Prostate
Rectum
Renal CC
Renal CH
Renal PA
Skin
Stomach
Testis
Thyroid
Uterus CS
Uterus EC
b
MFN2
MFN2 gene expression
8000
6000
4000
2000
0
Normal
Tumor
Adrenal
AML
Bladder
Breast
Colon
Esoph.
Liver
Lung AC
Lung SC
Ovary
Pancreas
Prostate
Rectum
Renal CC
Renal CH
Renal PA
Skin
Stomach
Testis
Thyroid
Uterus CS
Uterus EC
c
MFN1
P = 2.18e-06
MFN1 gene expression
2500
2000
1500
1000
500
Normal
Tumor
d
MFN2
P = 6.86e-03
MFN2 gene expression
1500
1000
500
Normal
Tumor
e
MFN1 (207098_s_at)
HR = 1.22 (1.11 − 1.35)
logrank P = 8.9e−05
Probability
Expression
low
high
Time (months)
f
MFN2 (201155_s_at)
HR = 0.8 (0.72 − 0.88)
logrank P = 1.4e−05
Probability
Expression
low
high
Time (months)

Supplementary Figure 1: MFNs expression is changed in breast cancer tissue

(a) TNM plot of the MFN1 expression across all tissues in available normal and tumor RNA sequencing data (n=3691 normal, 29376 tumor samples, *P<0.05). Data are resorted from https://tnmplot.com.

(b) TNM plot of the MFN2 expression across all tissues in available normal and tumor RNA sequencing data (n=3691 normal, 29376 tumor samples, *P<0.05). Data are resorted from https://tnmplot.com.

(c) Violin plot of the MFN1 expression in breast invasive carcinoma from RNA sequencing data available. Data are resorted from https://tnmplot.com.

(d) Violin plot of the MFN2 expression in breast invasive carcinoma from RNA sequencing data available. Data are resorted from https://tnmplot.com.

(e) Kaplan-Meier curve for breast cancer patients with high or low MFN1 levels. Data are resorted from Kaplan-Meier Plotter (http://kmplot.com). (Affymetrix id: 207098_s_at; Status: all. Follow up threshold: all; split on median.

(f) Kaplan-Meier curve for breast cancer patients with high or low MFN2 levels. Data are resorted from Kaplan-Meier Plotter (http://kmplot.com). Affymetrix id: 216205_s_at; Status: all. Follow up threshold: all; split on median.

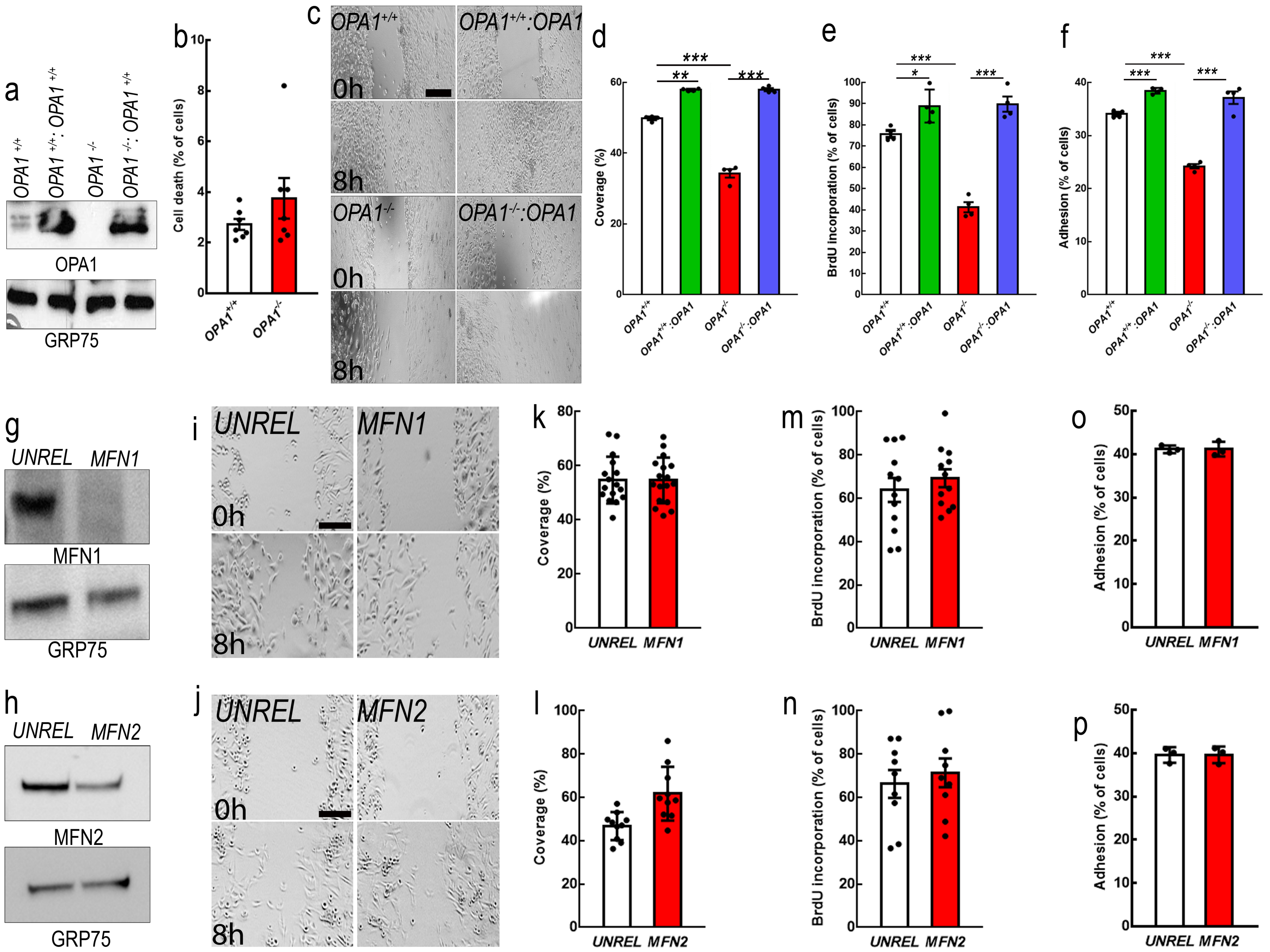

a
OPA1 +/+
OPA1 +/+: OPA1 +/+
OPA1 -/-
OPA1 -/-: OPA1 +/+
OPA1
GRP75
b
Cell death (% of cells)
OPA1+/+
OPA1-/-
c
OPA1+/+
OPA1+/+:OPA1
OPA1-/-
OPA1-/-:OPA1
0h
8h
d
Coverage (%)
e
BrdU incorporation (% of cells)
f
Adhesion (% of cells)
OPA1+/+
OPA1+/+:OPA1
OPA1-/-
OPA1-/-:OPA1
g
UNREL
MFN1
MFN1
GRP75
h
UNREL
MFN2
MFN2
GRP75
i
UNREL
MFN1
0h
8h
j
UNREL
MFN2
0h
8h
k
Coverage (%)
UNREL MFN1
l
Coverage (%)
UNREL MFN2
m
BrdU incorporation (% of cells)
UNREL MFN1
n
BrdU incorporation (% of cells)
UNREL MFN2
o
Adhesion (% of cells)
UNREL MFN1
p
Adhesion (% of cells)
UNREL MFN2

Supplementary Figure 2: MFNs are not required for breast cancer cells migration, proliferation and adhesion

(a) Quantification of cell apoptosis of MDA-MB-231 with the indicated genotype determined by annexin V/propidium iodide label by flow cytometry. n=4 independent experiments.

(b) Equal amounts of protein from breast cancer cells MDA-MB-231 with the indicated genotype were separated by SDS-PAGE and immunoblotted with the indicated antibodies.

(c) Representative brightfield images acquired at the indicated time points of MDA-MB-231 with the indicated genotype and transfected as indicated in a scratch-wound assay. Scale bar: 250 µm.

(d) Quantification of cell migration after 6h, experiments as in **(c)**. n=4 independent experiments. ***: p<0.0001.

(e) Quantification of proliferation of MDA-MB-231 with the indicated genotype and transfected as indicated determined by BrdU incorporation. n=12 independent experiments. ***: p<0.0001; *: p<0.05.

(f) Quantification of cell adhesion on fibronectin for 1h of MDA-MB-231 with the indicated genotype and transfected as indicated. n= 4 independent experiments. ***: p<0.0001.

(g-h) Equal amounts of protein from breast cancer cells MDA-MB-231 transfected for 72h with the indicated siRNA were separated by SDS-PAGE and immunoblotted with the indicated antibodies.

(i-j) Representative brightfield images acquired at the indicated time points of MDA MB-231 transfected with the indicated siRNA in a scratch-wound assay. Scale bar: 250 µm.

(k-l) Quantification of cell migration after 6h, experiments as in (**i-j**). n=16 (MFN1), n=9 (MFN2) independent experiments.

(m-n) Quantification of proliferation of MDA-MB-231 transfected with the indicated siRNA determined by BrdU incorporation. n=12 (MFN1), n=9 (MFN2) independent experiments.

(o-p) Quantification of cell adhesion on fibronectin for 1h of MDA-MB-231 cells transfected with the indicated siRNA for 72h. n= 4 independent experiments.

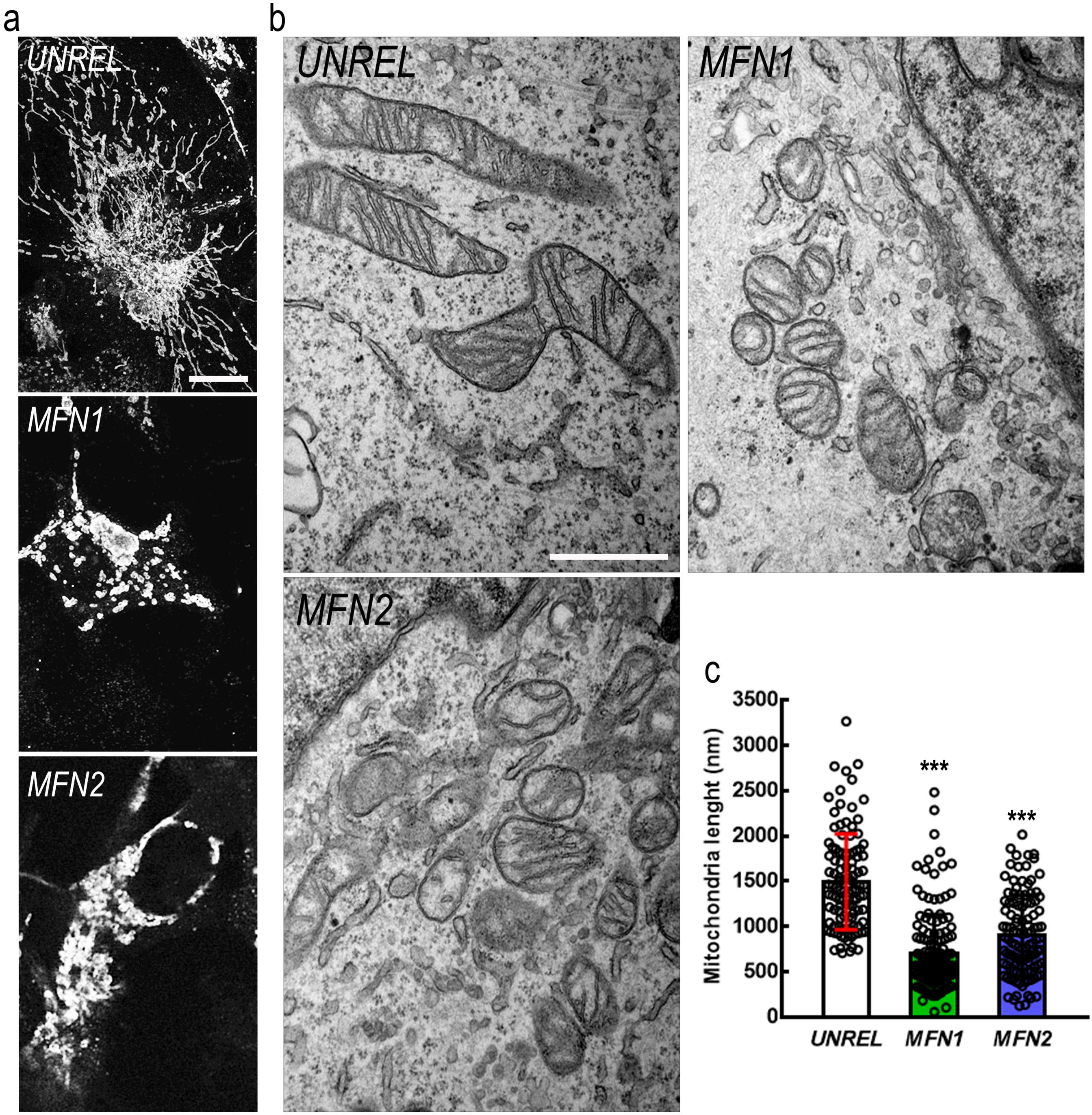

a
UNREL
MFN1
MFN2
b
UNREL
MFN1
MFN2
c
Mitochondria lenght (nm)
3500
3000
2500
2000
1500
1000
500
0
***
***
UNREL
MFN1
MFN2

Supplementary Figure 3: MFNs ablation induce mitochondrial fragmentation

(a) Representative confocal images of mitochondrial morphology in MDA-MB-231 transfected with the indicated siRNA for 72h and stained for TOM20. Scale bar: 30 μm.

(b) Representative EM images of MDA-MB-231 transfected for 72h with the indicatedsiRNA. Scale bar: 500 nm.

(c) Quantification of mitochondrial length in experiments as in **(B)**. n=240 mitochondria/condition from 3 independent experiments. *** p<0.001.

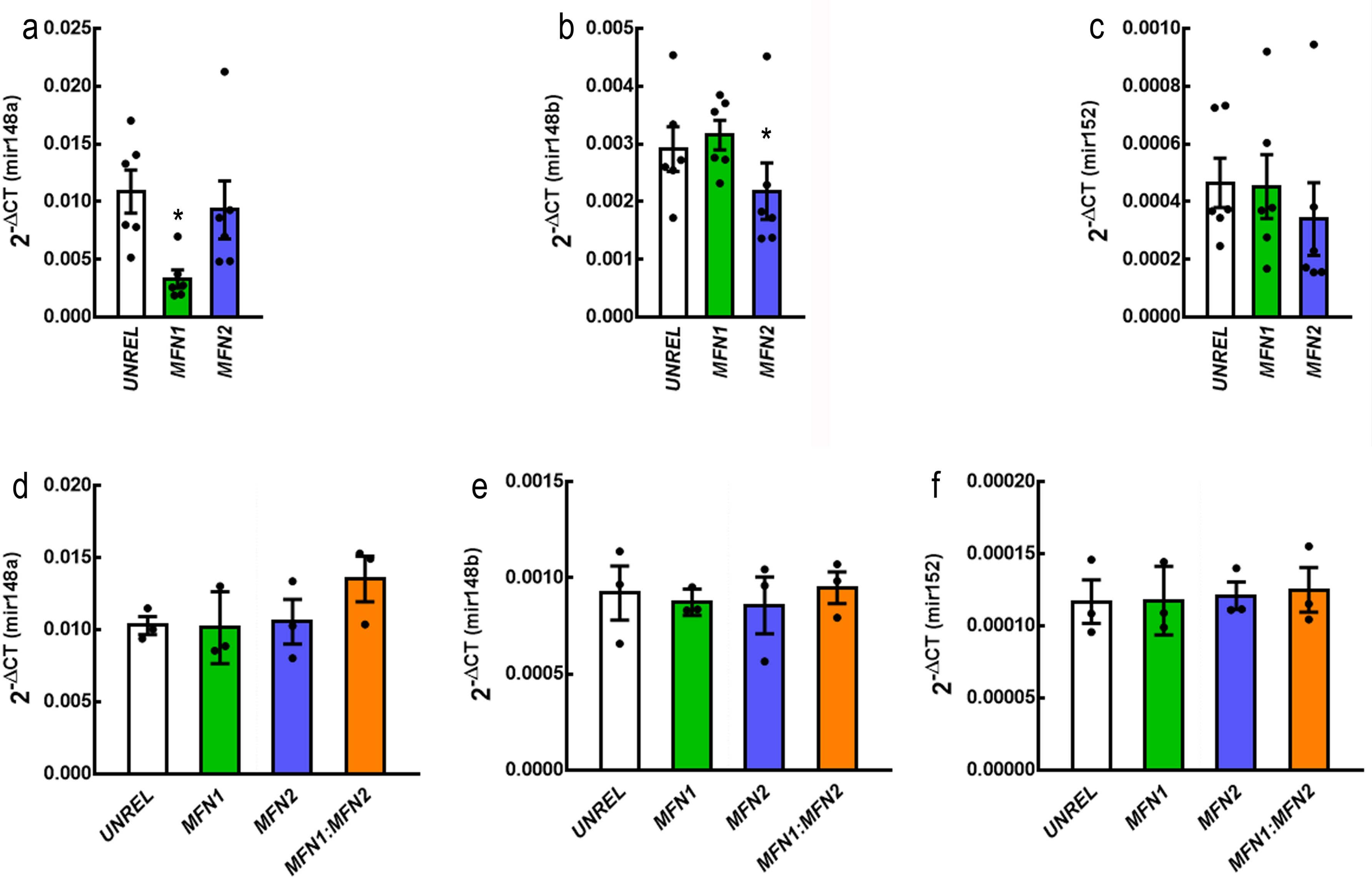

a
b
c
d
e
f
2-ΔCT (mir148a)
2-ΔCT (mir148b)
2-ΔCT (mir152)
0.025
0.020
0.015
0.010
0.005
0.000
0.005
0.004
0.003
0.002
0.001
0.000
0.0010
0.0008
0.0006
0.0004
0.0002
0.0000
0.0015
0.0010
0.0005
0.0000
0.00020
0.00015
0.00010
0.00005
0.00000
*
UNREL
MFN1
MFN2
MFN1:MFN2

Supplementary Figure 4: MFNs level does not affect miRNA 148a; 148b and 152 level.

(a) $2^{-\Delta ct}$ of *mir148a* levels determined by qRT-PCR in MDA-MB-231 transfected as indicated for 72h. n=6 independent experiments.

(b) $2^{-\Delta ct}$ of *mir148b* levels determined by qRT-PCR in MDA-MB-231 transfected as indicated for 72h. n=6 independent experiments.

(c)$2^{-\Delta ct}$ of *mir152* levels determined by qRT-PCR in MDA-MB-231 transfected as indicated for 72h. n=6 independent experiments.

(d) $2^{-\Delta ct}$ of *mir148a* levels determined by qRT-PCR in MDA-MB-231 with the indicated genotype. n=3 independent experiments.

(e) $2^{-\Delta ct}$ of *mir148b* levels determined by qRT-PCR in MDA-MB-231 with the indicated genotype. n=3 independent experiments.

(f) $2^{-\Delta ct}$ of *mir152* levels determined by qRT-PCR in MDA-MB-231 with the indicated genotype. n=3 independent experiments.

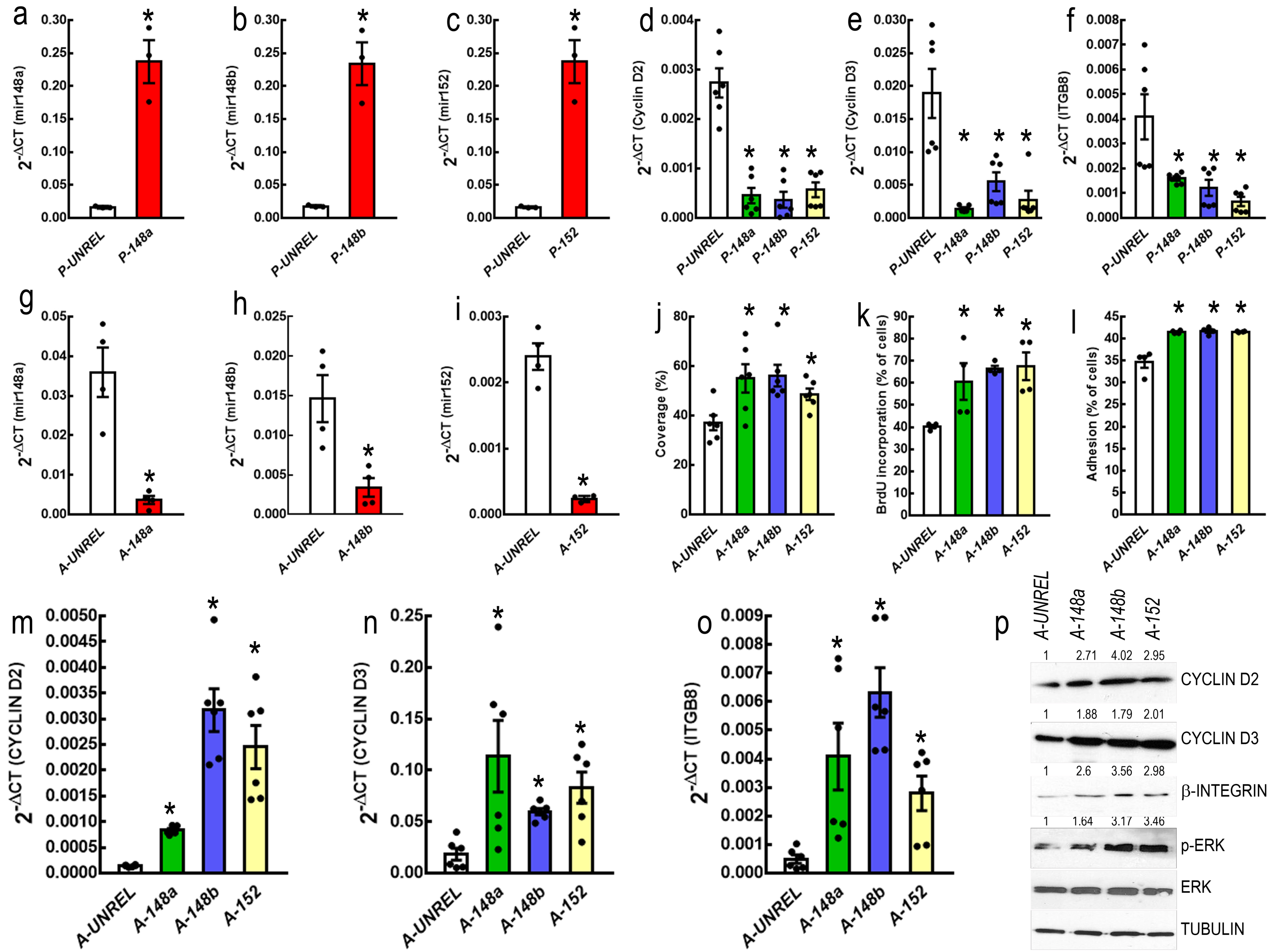

a
b
c
d
e
f
g
h
i
j
k
l
m
n
o
p
2-ΔCT (mir148a)
2-ΔCT (mir148b)
2-ΔCT (mir152)
2-ΔCT (Cyclin D2)
2-ΔCT (Cyclin D3)
2-ΔCT (ITGB8)
Coverage (%)
BrdU incorporation (% of cells)
Adhesion (% of cells)
2-ΔCT (CYCLIN D2)
2-ΔCT (CYCLIN D3)
2-ΔCT (ITGB8)
P-UNREL
P-148a
P-148b
P-152
A-UNREL
A-148a
A-148b
A-152
1 2.71 4.02 2.95
CYCLIN D2
1 1.88 1.79 2.01
CYCLIN D3
1 2.6 3.56 2.98
β-INTEGRIN
1 1.64 3.17 3.46
p-ERK
ERK
TUBULIN

Supplementary Figure 5: miRNA 148a, 148b and 152 level modulation regulates MDA-MB-231 migration, proliferation and adhesion

(a) $2^{-\Delta ct}$ of *mir148a* levels determined by qRT-PCR in MDA-MB-231 transfected as indicated for 24h. n=3 independent experiments. * p<0.05. P-= mimic miRNA.

(b) $2^{-\Delta ct}$ of *mir148b* levels determined by qRT-PCR in MDA-MB-231 transfected as indicated for 24h. n=3 independent experiments. * p<0.05. P-= mimic miRNA.

(c) $2^{-\Delta ct}$ of *mir152* levels determined by qRT-PCR in MDA-MB-231 transfected as indicated for 24h. n=3 independent experiments. * p<0.05. P-= mimic miRNA.

(d) $2^{-\Delta ct}$ of *Cyclin D2 mRNA* levels determined by qRT-PCR in MDA-MB-231 transfected for 24h with the indicated mimic miRNA. n=6 independent experiments. P-= mimic miRNA.

(e) $2^{-\Delta ct}$ of *Cyclin D3 mRNA* levels determined by qRT-PCR in MDA-MB-231 transfected for 24h with the indicated mimic miRNA. n=6 independent experiments. P-= mimic miRNA.

(f) $2^{-\Delta ct}$ of *ITGB8 mRNA* levels determined by qRT-PCR in MDA-MB-231 transfected for 24h with the indicated mimic miRNA. n=6 independent experiments. P-= mimic miRNA.

(g) $2^{-\Delta ct}$ of *mir148a* levels determined by qRT-PCR in MDA-MB-231 transfected as indicated for 24h. n=3 independent experiments. * p<0.05.. A-: anti-miRNA.

(h) $2^{-\Delta ct}$ of *mir148b* levels determined by qRT-PCR in MDA-MB-231 transfected as indicated for 24h. n=3 independent experiments. * p<0.05.. A-: anti-miRNA.

(i) $2^{-\Delta ct}$ of *mir152* levels determined by qRT-PCR in MDA-MB-231 transfected as indicated for 24h. n=3 independent experiments. * p<0.05.. A-: anti-miRNA.

(j) Quantification of cell migration of MDA-MB-231 transfected for 24h with the indicated anti-miRNA after 6h in a scratch wound assay. n=6 independent experiments. *: p<0.05. A-: anti-miRNA.

(k) Quantification of proliferation of MDA-MB-231 transfected for 24h as indicated determined by BrdU incorporation. n=6 independent experiments. *: p<0.05. A-: anti-miRNA.

(l) Quantification of cell adhesion on fibronectin of MDA-MB-231 transfected for 24h as indicated. n=4 independent experiments. *: p<0.05. A-: anti-miRNA.

(m) $2^{-\Delta ct}$ of *Cyclin D2 mRNA* levels determined by qRT-PCR in MDA-MB-231 transfected for 24h with the indicated anti-miRNA. n=6 independent experiments. A-: Anti-miRNA.

(n) $2^{-\Delta ct}$ of *Cyclin D3 mRNA* levels determined by qRT-PCR in MDA-MB-231 transfected for 24h with the indicated anti-miRNA. n=6 independent experiments. A-: Anti-miRNA.

(o) $2^{-\Delta ct}$ $2^{\Delta ct}$ of *ITGB8 mRNA* levels determined by qRT-PCR in MDA-MB-231 transfected for 24h with the indicated anti-miRNA. n=6 independent experiments. A-: Anti-miRNA.

(p) Equal amounts of protein from breast cancer cells MDA-MB-231 transfected for 24h with the indicated anti-miRNA were separated by SDS-PAGE and immunoblotted with the indicated antibodies. Numbers above each panel represent densitometry analysis. A-: Anti-miRNA.

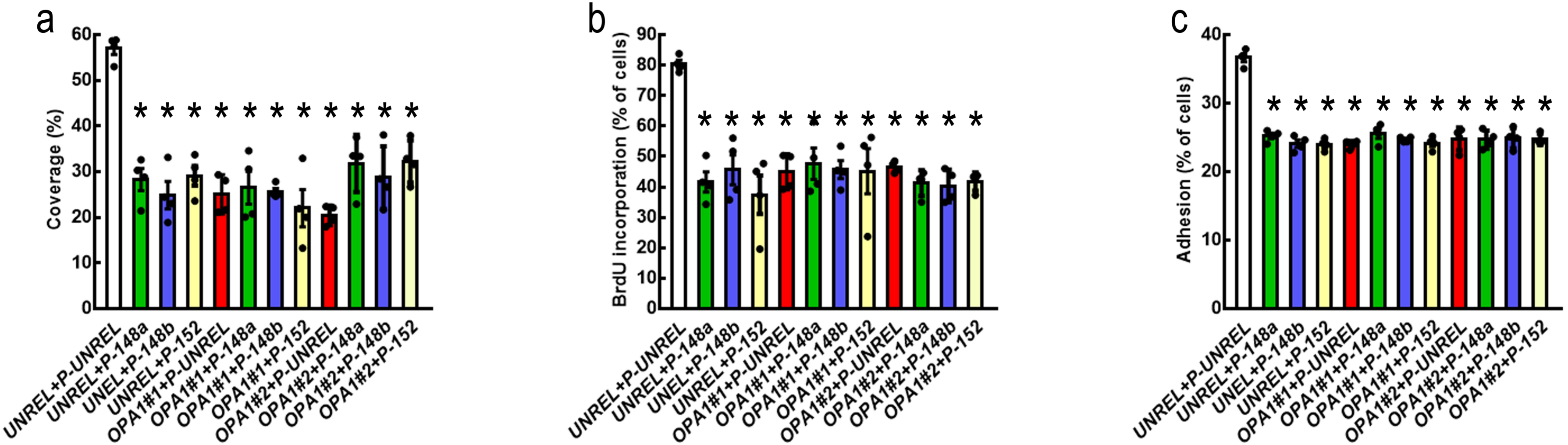
a
Coverage (%)
0
10
20
30
40
50
60
UNREL+P-UNREL
UNREL+P-148a
UNEL+P-148b
UNREL+P-152
OPA1#1+P-UNREL
OPA1#1+P-148a
OPA1#1+P-148b
OPA1#1+P-152
OPA1#2+P-UNREL
OPA1#2+P-148a
OPA1#2+P-148b
OPA1#2+P-152
b
BrdU incorporation (% of cells)
0
10
20
30
40
50
60
70
80
90
UNREL+P-UNREL
UNREL+P-148a
UNEL+P-148b
UNREL+P-152
OPA1#1+P-UNREL
OPA1#1+P-148a
OPA1#1+P-148b
OPA1#1+P-152
OPA1#2+P-UNREL
OPA1#2+P-148a
OPA1#2+P-148b
OPA1#2+P-152
c
Adhesion (% of cells)
0
10
20
30
40
UNREL+P-UNREL
UNREL+P-148a
UNEL+P-148b
UNREL+P-152
OPA1#1+P-UNREL
OPA1#1+P-148a
OPA1#1+P-148b
OPA1#1+P-152
OPA1#2+P-UNREL
OPA1#2+P-148a
OPA1#2+P-148b
OPA1#2+P-152

Supplementary Figure 6: No additive effect of OPA1 ablation and miRNA 148a, 148b and 152 overexpression.

(a) Quantification of migration of MDA-MB-231 transfected with the indicated siRNA for 72h and with the indicated miRNA for 24h by scratch wound assay after 6h. n= 4 independent experiments. *: $p<0.05$. P-: miRNA mimic.

(b) Quantification of proliferation of MDA-MB-231 transfected with the indicated siRNA for 72h and with the indicated miRNA for 24h determined by BrdU incorporation. n=4 independent experiments. *: $p<0.05$. P-: miRNA mimic.

(c) Quantification of cell adhesion on fibronectin of MDA-MB-231 transfected with the indicated siRNA for 72h and with the indicated miRNA for 24h. n=4 independent experiments. *: $p<0.05$. P-: miRNA mimic.
